# CXCL10 Promotes Spinal Macrophage Recruitment via the JAK/STAT3 Pathway to Induce Pain in Experimental Autoimmune Prostatitis

**DOI:** 10.1111/cpr.13784

**Published:** 2024-12-24

**Authors:** Lei Chen, Ziqi Chen, Jia Chen, Hexi Du, Xianguo Chen, Jing Chen, Hui Wang, Chaozhao Liang

**Affiliations:** ^1^ Department of Urology The First Affiliated Hospital of Anhui Medical University Hefei Anhui China; ^2^ Institute of Urology Anhui Medical University Hefei Anhui China; ^3^ Anhui Province Key Laboratory of Urological and Andrological Diseases Research and Medical Transformation Hefei Anhui China

**Keywords:** chronic prostatitis, CXCL10, nerve growth factor, pain, spinal macrophages, STAT3

## Abstract

The aim is to explore the mechanisms underlying pain development in chronic prostatitis and identify therapeutic targets for pain management in patients with chronic prostatitis. RNA sequence of the spinal cord dorsal horns and proteomic analysis of spinal macrophages of experimental autoimmune prostatitis (EAP) mice were conducted to identify pain‐related genes, proteins and signalling pathways. The clodronate liposome, CXCR3 and P‐STAT3 inhibitors, NGF antibody and cromolyn sodium were used to investigate the roles of the CXCL10/CXCR3, JAK/STAT3 and NGF/TrKA pathways in spinal macrophage recruitment and pain response. Finally, prostate tissues from benign prostate hyperplasia (BPH) patients were collected to validate the aforementioned results. Neuron and astrocyte‐derived CXCL10 was associated with spinal macrophage recruitment, and CXCL10/CXCR3 axis could regulate the chemotaxis of macrophage to the spinal cord in EAP mice. Results of proteomic analysis found that CXCL10 could regulate the JAK/STAT3 pathway to mediate neuroinflammation in EAP, which was validated in vivo and in vitro experiments. The number of mast cells and expressions of NGF, TrKA and PGP9.5 increased in the prostates of EAP mice and BPH patients, and targeting NGF could reduce spinal macrophage recruitment and pain response. NGF was the triggering factor to induce chemotaxis of spinal macrophages and neuroinflammation, and the CXCL10/CXCR3 axis and JAK/STAT3 pathway was involved in spinal macrophage recruitment and infiltration, which provided therapeutic targets for pain management.

## Graphical abstract

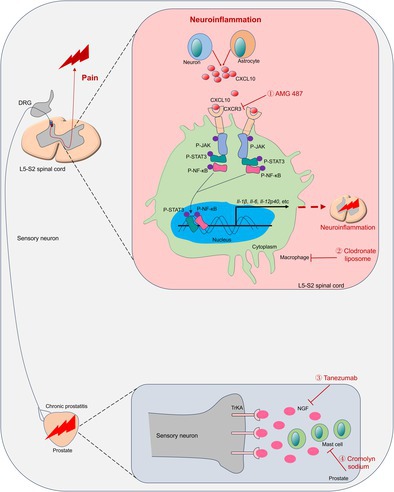

**Abbreviations**
BPH: benign prostate hyperplasia
CFA: complete Freund's adjuvant
CXCL10: C‐X‐C chemokine 10
DEGs: differentially expressed genes
DRG: dorsal root ganglia
EAP: experimental autoimmune prostatitis
ESWT: extracorporeal shock wave therapy
FAHAMU: First Affiliated Hospital of Anhui Medical University
GSEA: Gene Set Enrichment Analysis
JAK: Janus kinase
LPS: lipopolysaccharides
NGF: nerve growth factor
NOD: non‐obese diabetic
P2X4R: P2X4 receptor
PAgs: prostate antigens
SCDH: spinal cord dorsal horn
STAT: signal transducers and activator of transcription
WGCNA: Weighted Gene Co‐Expression Network Analysis

## 1 Introduction

As one of the most common diseases in young men in the urology department, chronic prostatitis severely reduces the quality of life [1]. Symptoms including pelvic pain, sexual dysfunction and voiding are the main complaints of patients, and men with long‐term chronic prostatitis tend to have depression behaviours [2]. Depression could in turn aggravate pain and lead to deterioration of quality of life [3]. Currently, therapeutic approaches for improving the quality of life of chronic prostatitis patients have been developed, including antibiotics, phytotherapy, acupuncture, α‐blockers, extracorporeal shock wave therapy (ESWT), and so forth [2, 4, 5]; however, the therapeutic efficacies remain unsatisfactory. Therefore, deepening the mechanisms underlying the pain response in chronic prostatitis was urgent.

It is reported that neuroinflammation occurs in chronic pain conditions, and neuroinflammation is involved in the chronicity of pain [6]. Neuroinflammation belongs to the local inflammation that occurs in the peripheral and central nervous systems, and infection and autoimmune conditions are the triggering factors. Activation of infiltrated leucocytes and glial cells, elevated immune mediator levels, and changes in the permeability of blood vessels are the characterisations of neuroinflammation [6, 7]. In response to bacterial infection and tissue injury, inflammatory substances including LPS, HMGB1, and so forth, could activate the primary sensory neurons to induce glial cell activation and pro‐inflammatory cytokines secretion, which cause neuroinflammation, synaptic plasticity and pain development [8]. In chronic prostatitis, microglia and astrocytes were associated with pain through the release of pro‐inflammatory cytokines in the spinal cord [9]. In addition, the Notch pathway was found to promote microglia activation and cytokine release to induce pain in chronic prostatitis [10]. Hence, the roles of glial cell activation and cytokine release may play an important in pain in chronic prostatitis, which deserves further investigation.

Macrophages could regulate immune function and pain response, and macrophage‐derived TNF‐α and IL‐1β could promote pain transduction and conduction [11]. Microglia are tissue‐resident macrophages in the spinal cord and brain, and spinal microglia/macrophages are demonstrated to be engaged in the initiation of neuropathic pain [8, 12, 13]. Macrophages in the dorsal root ganglia (DRG) could promote the development of mechanical hypersensitivity in neuropathic pain, which was also involved in the maintenance of pain response, and the elimination of macrophages attenuated the mechanical hypersensitivity [14]. In the nerve injury model, the P2X4 receptor (P2X4R) was highly expressed in spinal microglia, and inhibition of P2X4R attenuated tactile allodynia, and intraspinal administration of pre‐hyperactivated microglia induced tactile allodynia in naïve animals [15]. Taken together, spinal macrophages were involved in pain response, while their roles in chronic prostatitis remained unclear.

Non‐obese diabetic (NOD) mice were genetically susceptible to autoimmune conditions, including adrenalitis, Type 1 diabetes, thyroiditis and prostatitis [16, 17, 18]. In chronic prostatitis, NOD mice were used to establish the experimental autoimmune prostatitis (EAP) model, and the EAP mice exhibited the main characteristics of chronic prostatitis, including (1) pelvic pain symptoms, (2) prostate inflammation and (3) increased cytokines levels [17, 19]. Based on the good performance of the EAP model by using NOD mice, this established model was widely adopted to explore the pathogenesis underlying chronic prostatitis [19].

The C‐X‐C chemokine 10 (CXCL10) is one member of the chemokine family, and in spinal nerve ligation, the expression levels of CXCL10 and CXCR3 were increased in DRG neurons, and the p38 and ERK pathway are downstream of the CXCL10/CXCR3 axis to induce neuropathic pain [20]. Moreover, the CXCL10/CXCR3 axis also contributes to trigeminal neuropathic pain and chronic constriction injury‐related pain [21, 22]. CCL2 was demonstrated to be associated with prostate, DRG and spinal cord macrophage infiltration in the EAP model [23]. Our previous study showed that serum CXC10 levels were positively associated with symptoms of chronic prostatitis, and the CXCL10/CXCR3 axis could activate the p38 and ERK to induce prostate macrophage migration and cytokine release [24]. Based on the association between CXCL10 and pain in chronic prostatitis, we proposed that the elevated CXCL10 may attract macrophages to the spinal cord to induce neuroinflammation and pain in chronic prostatitis.

In this study, we found that the CXCL10/CXCR3 axis was involved in spinal macrophage infiltration and pain development, and the JAK/STAT3 pathway was downstream of the CXCL10/CXCR3 axis to induce the migration and cytokine release of spinal macrophages. Moreover, the nerve growth factor (NGF)/TrKA axis was associated with the recruitment of spinal macrophages and pain development in chronic prostatitis, and inhibition of NGF decreased spinal macrophage recruitment and attenuated pain response in chronic prostatitis. This study demonstrated the important roles of the CXCL10/CXCR3, JAK/STAT3 and NGF/TrKA pathways in neuroinflammation and pain in chronic prostatitis, which provided novel therapeutic targets for pain management in chronic prostatitis.

## 2 Materials and Methods

### 2.1 Establishment of Experiment Autoimmune Prostatitis Model and Treatments

**FIGURE 1:**
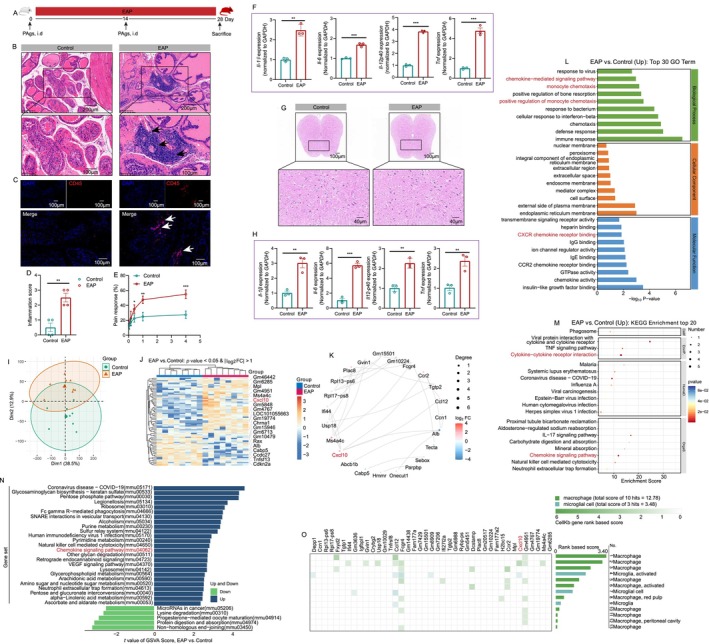
Identification of neuroinflammation and DEGs in the spinal cord of EAP and control mice. The whole process of EAP induction (A). Results of H&E and CD45 staining displayed the number of lymphocyte infiltration in the prostate of the control and EAP group (B and C), and the inflammation score between the control and EAP groups (D). Results of the behavioural test showed the response rate between mice in the control and EAP groups (E). The expression levels of immune mediators including *Il‐1β*, *Il‐6*, *Il‐12p4*0 and *Tnf* mRNA in the prostate of control and EAP mice (F). H&E staining showed the morphology of the spinal cord of control and EAP mice (G). The expression levels of immune mediators including *Il‐1β*, *Il‐6*, *Il‐12p40* and *Tnf* mRNA in the spinal cord of control and EAP mice (H). PCA analysis based on the results of RNA sequencing (I). The heatmap displayed the expression levels of the DEGs (J). The PPI showed the relationship among these DEGs (K). The function of the identified DEGs was annotated with the GO terms and KEGG enrichments (L and M). GSVA analysis based on the identified DEGs (N). The Cellkb database displayed the relationship between identified DEGs and macrophage/microglia activation (O). DEGs: differentially expressed genes; EAP: experimental autoimmune prostatitis; i.d.: intradermally; PAgs: prostate antigens; PCA: principal component analysis; PPI: protein‐protein interaction. ** *p* < 0.01; *** *p* < 0.001.

The EAP model was established following previous studies [19, 25]. For prostate antigens (PAgs) extraction, prostate glands isolated from Sprague–Dawley rats were homogenised in phosphate‐buffered saline (0.01 M, pH 7.2) with protease inhibitors and Triton X‐100 in the homogeniser (KZ‐5F‐3D, Servicebio). After centrifuging at 10,000 *g* × 30 min at 4°C, the protein concentration of the PAgs was detected with a BCA assay kit and stored at −80°C. PAgs (300 μg/mouse) were emulsified with complete Freund's adjuvant (CFA, Sigma‐Aldrich) to immunise the NOD mice (Figure 1A). Six weeks of NOD mice were purchased from the Nanjing Biomedical Research Institute of Nanjing University (Nanjing, China). The emulsion was injected at four sites, including the tail base (50 μL), the right and left foot pad (25 μL, respectively), and the shoulder (50 μL). The control mice were immunised with the same volume (150 μL) of PBS‐emulsified CFA. For drug treatment, AMG487 (a CXCR3 antagonist) was dissolved in 40% DMSO and intrathecally injected into NOD mice (100 μg/mouse) [22]. For tanezumab (an NGF antibody, GC69986, GLP) administration, tanezumab was intraperitoneally (ip) injected into NOD mice plus once injection into the prostates (100 μg/mouse) [26], and cromolyn sodium (mast cell stabiliser, HY‐B0320A, MCE) was ip injected to NOD mice (0.5 mg/kg) for 14 days [27]. This study was conducted under the approval of the Committee for Animal Care and Use of the Animal Center of Anhui Medical University (Approval No. LLSC20232178).

### 2.2 Behavioural Tests

Pain response was assessed as previously described [25, 28]. Briefly, after acclimating for 30 min in the chamber, and the mice were tested for pelvic pain by using von Frey filaments. The filament was applied to the lower abdominal area. The filament forces of 0.04, 0.16, 0.4, 1.0 and 4.0 g were applied in each mouse, respectively (10 times in total), and the assessment was performed in different areas with the lower abdominal area. Positive pain responses were defined as follows: (1) sharp retraction of the abdomen; (2) jumping; and (3) licking or scratching of the tested area, and times of positive response/10 × 100% were defined as the positive response rate.

### 2.3 Haematoxylin and Eosin (H&E) Staining

The human and NOD mice prostates were fixed, dehydrated and embedded. Then, the paraffin‐embedded 4 μm tissues were treated with xylene and alcohol, and after washing with distilled water, the tissues were stained with H&E. The pictures were obtained under the slide scanner (3DHISTECH, Pannoramic MIDI). The prostate inflammation in EAP mice was defined as four levels as previously described [29].

### 2.4 Immunohistochemistry

As described in H&E staining, the tissues were deparaffinised and rehydrated. Then, antigen retrieval with citrate buffer and destroy endogenous peroxidase with 3% H_2_O_2_ were performed. The non‐specific sites were blocked with goat serum. Antibody against NGF was incubated overnight at 4°C, and the tissues were incubated with secondary antibodies. The cell nucleus was counterstained with haematoxylin. The images were obtained under a light microscope.

### 2.5 Immunofluorescence

Samples were prepared as the abovementioned processes in H&E staining, and antibodies against phosphorylated sites of JAK1 at Tyr1022 (P‐JAK1), JAK2 at Tyr1007 (P‐JAK2), NF‐κB at Ser536 (P‐NF‐κB) and STAT3 at Tyr705 (P‐STAT3) were used to detect the phosphorylation levels of P‐JAK1, P‐JAK2, NF‐κB and STAT3. The tissues and cell slides were fixed and permeabilised, and the non‐specific sites were blocked with goat serum. Then, the tissues were incubated with the primary antibodies against P‐JAK1, P‐JAK2, P‐NF‐κB and P‐STAT3 overnight at 4°C. Afterwards, secondary antibodies were incubated for 2 h. Finally, the tissues were stained with DAPI, and the immunofluorescent images were obtained under confocal laser scanning microscopy (LSM 800, ZEISS, German). Antibodies information can be found in Table S1.

### 2.6 Toluidine Blue Staining

As described in H&E staining, the tissues were deparaffinised and rehydrated, and then incubated in 0.02% toluidine blue solution for 10 min. After being washed and fixed, the images were obtained under a light microscope.

### 2.7 Cell Culture and Treatments

The RAW264.7 and iBMDM cell lines were purchased from Procell (Wuhan, China), which was cultured in Dulbecco's modified Eagle's medium with 10% fetal bovine serum and 1% penicillin/streptomycin at 37°C with 5% CO_2_. For cell treatment, AMG487 (2 μM, HY‐15319, MCE) and stattic (10 μM, HY‐13818, MCE) were used to pretreat the cells for 1 h, and then lipopolysaccharides (LPS, 100 ng/mL, L2880, Sigma) [30] and CXCL10 (10, 50 and 100 ng/mL, 250–16, PeproTech) [30] were used to treat the cells for 24 h.

### 2.8 RNA Extraction and RT‐qPCR

RNA was extracted by using TRIzol, and after measuring the RNA concentration, cDNA was synthesised by using a PrimeScript RT reagent Kit (RR047A, Takara). Real‐time PCR was performed with TB Green Premix Ex Taq kit (RR820A, Takara). Primers used in this study were presented in Table S2.

### 2.9 Western Blot

Western blot was performed as previously described, and antibodies against phosphorylated sites of JAK1 at Tyr1022 (P‐JAK1), JAK2 at Tyr1007 (P‐JAK2), NF‐κB at Ser536 (P‐NF‐κB) and STAT3 at Tyr705 (P‐STAT3) were used to detect the phosphorylation levels of JAK1, JAK2, NF‐κB and STAT3 [28]. Briefly, after extracting total proteins and measuring protein concentration, samples were boiled with a loading buffer. The sodium dodecyl sulfate‐polyacrylamide gel was conducted and proteins were transferred onto a polyvinylidene difluoride membrane. The membranes were blocked with 5% nonfat milk for 2 h and incubated with primary antibodies against CXCL10, CXCR3, JAK1, JAK2, NF‐κB, STAT3, P‐JAK1, P‐JAK2, P‐NF‐κB, P‐STAT3 and GAPDH overnight. After washing with western washing buffer, the membranes were incubated with secondary antibodies at room temperature. The bands were obtained under the Tanon system (Shanghai, China).

### 2.10 Migration Assay

The cells suspended in 200 μL serum‐free medium were seeded in the upper transwell chambers (Costar, Bodenheim), and stattic and AMG487 were added to the upper chambers for 1‐h pretreatment, and CXCL10 and LPS were added to the lower chambers with 600 μL serum‐free medium for 24 h. The migrated cells on the lower surface of the membrane were fixed. After staining with 0.05% crystal violet for 15 min, the images were obtained under a light microscope.

### 2.11 RNA Sequence

The spinal cord dorsal horn (SCDH) of EAP mice was obtained and stored at −80°C. The RNA sequencing was conducted by OE Biotech Co. Ltd. (Shanghai, China). Briefly, after extracting total RNA, and purified and quantifying by using a NanoDrop 2000 spectrophotometer (Thermo Scientific), the libraries were sequenced under the Illumina Novaseq 6000 platform according to the manufacturer's instruction of the VAHTS Universal V6 RNA‐seq Library Prep Kit. After processing the raw reads of a fastq format file with fastp [31] and deleting the reads with low quality, the data were further processed with HISAT2 [32]. Gene expression form was processed into Fragments Per Kilobase Million (FPKM) [33], and gene expression count data were obtained by using HTSeq‐count [34].

### 2.12 Identification of Differentially Expressed Genes (DEGs) Between EAP and Control Groups

RNA sequence count data were used to identify DEGs between EAP and control groups by using DESeq2 packages in R software. To illustrate the potential function of identified DEGs, GO [35], KEGG [36], Gene Set Enrichment Analysis (GSEA) [37], Reactome and WikiPathways enrichment analysis were performed based on the identified DEGs by using OECloud tools (https://cloud.oebiotech.com), R and GSEA software. The CellKb database included marker gene sets of different cell types across 10 species (https://www.cellkb.com), and we analysed the association between DEGs and macrophage/microglia activation.

### 2.13 Weighted Gene Co‐Expression Network Analysis (WGCNA)

WGCNA analysis was performed by using the RNA sequence data with the ‘WGCNA’ package in R software [38]. The gene expression variances between samples were calculated, and the top 25% of variance genes in 16 samples were included for WGCNA analysis. The ‘pickSoftThreshold’ function was used to identify soft threshold power *β* according to the scale‐free topology model fit (signed *R* ^2^) ≈0.9. The modules were identified by using hierarchical clustering and the dynamic tree cut, and the dynamic modules with high similarities were merged and the cluster dendrogram was used to visualise the modules with different colours. To generate reliable modules, the minimal number of genes and the threshold to merge similar modules were set to 30 and 0.25, respectively. The module‐trait analysis was also performed, and the gene module with *p* < 0.05 was identified as an EAP‐related gene module, and the gene list was extracted for further analysis. To further screen EAP‐related genes, the results of DESeq2 and WGCNA analyses were intersected and visualised by a Venn diagram.

### 2.14 Isolation of F4/80^+^ Cells in the Spinal Cord

The spinal macrophages were isolated from mice in EAP and EAP + AMG487 groups. Briefly, the spinal cord of the NOD mice was isolated and mechanically and enzymatically digested in DMEM medium with collagenase (1 mg/mL, 10269638001, Roche), DNase I (10 mg/mL, 10104159001, Roche), papain (5 U/mL, LS003119, Worthington) and EDTA (0.5 M, 15575020, Invitrogen) at 37°C [39]. Then, the suspensions were filtered through the 70 μm filter to remove cell clumps and washed with buffer, which was centrifugated and resuspended with buffer and stained with anti‐F4/80 microbeads ultrapure reagent at 4°C for 15 min by using the Anti‐F4/80 MicroBeads UltraPure (130–110‐443, Miltenyi Biotec), and the suspensions were centrifugated at 300 *g* × 5 min. The cells were resuspended in 500 μL buffer and added to the LS columns in the magnetic field. After passing through the unlabelled cells, the LS columns were placed on a collecting and then pushed the plunger into the columns to flush out the labelled cells in a collection tube, and the isolated cells were detected under the flow cytometry to identify the percentage of macrophages.

### 2.15 Proteomic Analysis

The isolated spinal macrophages were processed with lysis buffer with PMSF and subsequently sonicated on ice for 2 min, and protein concentration was detected with a BCA assay kit. The 50 μg of proteins were digested, briefly, DTT (5 mM) was added to the samples, which were incubated at 55°C for 30 min, and 10 mM iodoacetamide was added for 15 min. Then the samples were added with precooled acetone and placed at −20°C overnight. After centrifuging to collect the precipitation, the enzymolysis dilution was added to treat the samples at 37°C for 12 h. The digested samples were lyophilised and the digested peptides were desalted with SOLA SPE 96‐plate column. Liquid chromatography‐mass spectrometry was performed under a Tims TOF Pro2 mass spectrometer (Thermo Scientific) and an Easyspray source (Thermo Scientific). The data were researched by using the Spectronaut Pulsar 17.5 (Biognosys) with the UniProt‐ *Mus musculus* ‐10090‐2023.2.1 database. GSEA, WikiPathways and Rectome were used to annotate the differentially expressed protein. Protein–protein interaction (PPI) analysis was performed with an online tool (https://string‐db.org/).

### 2.16 Flow Cytometry

Cell suspensions were obtained from mice spleens, and after incubating with anti‐F4/80 and anti‐CD11b antibodies for 1 h at 4°C, cells were washed with PBS and detected by using a CytoFLEX flow cytometer (Beckman) in accordance with a previous study [28].

### 2.17 Clinical Specimen Collection

Benign prostatic hyperplasia (BPH) patients' prostate tissues were collected at the First Affiliated Hospital of Anhui Medical University (FAHAMU), which was following the Declaration of Helsinki Principles, and this study was approved by the ethical committee of FAHAMU. The exclusion criteria were as follows: patients with (1) incidental prostate cancer, (2) urinary retention, (3) urinary tract infection or (4) prostate intraepithelial neoplasia. Written informed consent was obtained from the patient. The criterion for evaluating prostate inflammation in BPH patients was in accordance with a previous study [40].

### 2.18 Statistical Analysis

GraphPad software (Version 8.3) and R software (Version 4.2) were used to analyse the data. All data were presented as means ± SEM, and a two‐tailed Student's *t*‐test was used to analyse the data between two groups, and the one‐way ANOVA with Tukey's multiple comparisons test was used to analyse the data among multiple groups. Parametric tests were used when data showed normal distribution and homogeneity of variance, and non‐parametric tests were used when data were not normal distribution or not homogeneity of variance. *p* < 0.05 was regarded as statistical significance. For immunofluorescence assay, the F4/80^+^/CD11b^+^, F4/80^+^/P‐JAK1^+^, F4/80^+^/P‐JAK2^+^ and P‐NF‐κB^+^/P‐STAT3^+^ cells were counted, and the difference in positive cell number between groups was compared. For RNA‐seq data analysis, the |log2|FC|| > 1 and *p* value < 0.05 were set to identify DEGs between control and EAP groups, and *p* value < 0.05 was set to identify EAP‐related modules in WGCNA analysis. For proteomic analysis, the |log2|FC|| > 1 and *p* value < 0.05 were set to identify differentially expressed proteins between EAP + AMG487 and EAP groups to identify pain‐related proteins.

## 3 Results

### 3.1 Identification of Neuroinflammation and DEGs in the L5‐S2 Spinal Cord of Control and EAP Mice

As shown in Figure 1A–C, the results of H&E and CD45 staining displayed that almost no lymphocyte was found in the prostate of the control group, while a great number of lymphocyte infiltration was detected in the EAP group, and compared to the control group, the inflammation score was higher in the EAP group (Figure 1D). Results of the behaviour test showed that the EAP mice had higher response rates than the control group (Figure 1E). The expression levels of immune mediators including *Il‐1β*, *Il‐6*, *Il‐12p4*0 and *Tnf* mRNA were increased in the prostate of EAP mice, which demonstrated the successfully established EAP model (Figure 1F). H&E staining showed the morphology of the L5‐S2 spinal cord of control and EAP mice (Figure 1G). The mRNA expression levels of immune mediators including *Il‐1β*, *Il‐6*, *Il‐12p40* and *Tnf* were increased in the spinal cord of EAP mice (Figure 1H). Previous studies found that the activated microglia and astrocytes in the spinal cord were associated with pain response in prostatitis [41, 42], and pain was relief and pain‐associated mediators including IL‐1β and BDNF were decreased after inhibition of the activated microglia [42], and spinal c‐*fos* expression was associated with central sensitisation in prostatitis model [43]. Therefore, neuroinflammation in the spinal cord was involved in chronic prostatitis pain, which deserves further exploration.

The DEGs were identified to investigate the potential mechanisms underlying chronic prostatitis pain. PCA analysis showed that based on the results of RNA sequencing, EAP samples were different from control samples (Figure 1I). In total, 70 DEGs were identified, and detailed information on these DEGs can be found in Table S3. The top 10 up‐regulated DEGs included *Cxcl10*, *Gm6285*, *Mpl*, *Gm5848*, *Ms4a4c*, *Gm46442*, *Gm4767*, *LOC101055663*, *Gm4951* and *Gm19774* (Figure S1A). The heatmap displayed the expression levels of the 70 DEGs (Figure 1J). The PPI showed the relationship among these DEGs (Figure 1K). Based on the identified DEGs, GO terms, KEGG and WikiPathways enriched ‘positive regulation of monocyte chemotaxis’, ‘Spinal cord injury’, and so forth (Figures S1B–D and S2A–C). As shown in Figure 1L,M, based on the up‐regulated DEGs, chemotaxis‐related terms attracted our attention, including ‘chemokine−mediated signalling pathway’, ‘CXCR chemokine receptor binding’, ‘spinal cord injury’, and so forth, which indicated that chemokines may play an important role in the development of neuroinflammation and pain in EAP. For the down‐regulated DEGs, the chemokine‐related pathways were not enriched, implying the important association between the up‐regulated DEGs and chemokines (Figure S3A–C). Results of GSVA also found that the chemokine signalling pathway was associated with EAP (Figure 1N). In addition, the GSEA results showed that microglial cell activation and microglia pathogen phagocytosis pathway were significantly associated with EAP (Figure S1C–D). The Cellkb database (https://www.cellkb.com/) was used to investigate the relationship between identified DEGs and macrophage/microglia activation, and the results showed that several genes including *Cxcl10*, *Ccr2* and *Ccl12*, and so forth, were associated with macrophage/microglia activity (Figure 1O). Based on the abovementioned findings, we proposed that chemokines may attract and activate the macrophage/microglia to induce neuroinflammation and pain, which deserved further validation in chronic prostatitis.

### 3.2 Neurons and Astrocytes Are Potential Sources of CXCL10 to Attract Macrophages to the Spinal Cord

**FIGURE 2:**
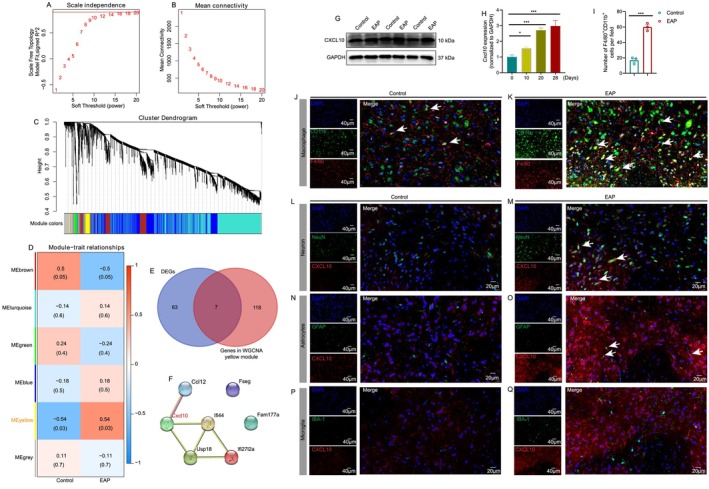
Neurons and astrocytes are potential sources of CXCL10 to attract macrophages to the spinal cord. The parameters of WGCNA analysis (A and B). The results of the WGCNA analysis identified EAP‐related modules (C and D). The common genes in the MEyellow module and the identified DEGs (E). PPI showed the relationship among these identified genes with WGCNA analysis (F). Results of western blotting analysis showed the CXCL10 protein levels in the spinal cord of control and EAP mice (G). The expression levels of spinal *Cxcl10* mRNA at 0, 10, 20 and 28 days of EAP induction (H). The number of macrophages in the spinal cord of control and EAP mice (I–K). The results of the immunofluorescence assay showed the expression of CXCL10 and its relationship with NeuN, GFAP and IBA‐1 (L–Q). DEGs: differentially expressed genes. EAP: experimental autoimmune prostatitis; PPI: protein–protein interaction; WGCNA: weighted gene co‐expression network analysis. * *p* < 0.05; *** *p* < 0.001.

To further screen EAP‐related genes, we performed WGCNA analysis to identify modules related to EAP. After filtering the last 75% of variance genes in 16 samples, 4022 genes were included for WGCNA analysis (Figure 2A,B). Our results showed that six modules were obtained, which were coloured blue, brown, green, grey, turquoise and yellow (Figure 2C,D). The genes included in these six modules are displayed in Table S4. As shown in Figure 2D, the module−trait relationship plot indicated that the MEyellow module was associated with EAP. The gene list in MEyellow modules was extracted and intersected with the identified 70 DEGs, and seven genes were selected, including *Cxcl10*, *Ccl12*, *Ifi44*, *Usp18*, *Ifi27l2a*, *Fseg* and *Fam177a* (Figure 2E), and the PPI showed the relationship among these genes (Figure 2F). Among these genes in Figure 2F, *Cxcl10* belonged to the chemokine family, and we proposed that *Cxcl10* may induce spinal macrophage infiltration and activation to cause neuroinflammation and pain response in chronic prostatitis.

Results of the western blot demonstrated that CXCL10 protein levels were higher in the spinal cord of EAP mice than in the control mice (Figure 2G). In addition, at 0, 10, 20 and 28 days, infiltrated lymphocytes in the prostates, inflammation score and pain response increased gradually (Figure S4A–D), and qPCR also found that the spinal *Cxcl10* mRNA levels were gradually elevated at 0, 10, 20 and 28 days (Figure 2H). The number of F4/80^+^CD11b^+^ cells was greater in the spinal cord of EAP mice than in the control mice (Figure 2I–K). Then, the immunofluorescence assay showed that CXCL10 was co‐localised with NeuN and GFAP, which were specific markers of neuron and astrocyte, respectively, indicating that neurons and astrocytes were potential sources of CXCL10 production in the spinal cord of EAP mice (Figure 2L–O). Conversely, CXCL10 was not detected in microglial cells (Figure 2P,Q). Therefore, neurons and astrocytes‐secreted CXCL10 may attract macrophages to the spinal cord to induce neuroinflammation in EAP mice.

### 3.3 Depletion of Macrophage Attenuates Spinal Macrophage Recruitment and Pain in EAP Mice

**FIGURE 3:**
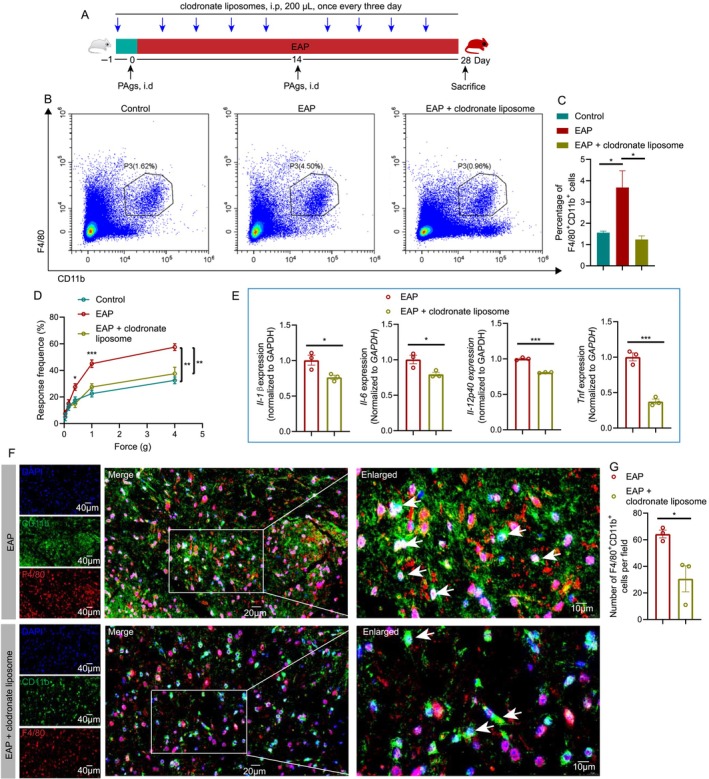
Depletion of macrophage suppresses spinal macrophage recruitment and pain in EAP mice. The whole process of clodronate liposome treatment in EAP mice (A). The results of flow cytometry showed the percentage of F4/80^+^CD11b^+^ cells in EAP mice treated with clodronate liposome (B and C). The pain responses in mice in control, EAP, EAP + clodronate liposome groups (D). The number of spinal F4/80^+^CD11b^+^ cells in EAP and EAP + clodronate liposome groups (E). The mRNA expression levels of *Il‐1β*, *Il‐6*, *Il‐12p4*0 and *Tnf* in EAP and EAP + clodronate liposome groups (F and G). EAP: Experimental autoimmune prostatitis. *p < 0.05.

The results of flow cytometry showed that clodronate liposome could effectively eliminate macrophages in EAP mice, and the percentage of F4/80^+^CD11b^+^ cells decreased in EAP mice treated with clodronate liposome (Figure 3A–C), and depletion of macrophages attenuated pain response in mice (Figure 3D). The *Il‐1β*, *Il‐6*, *Il‐12p4*0 and *Tnf* mRNA expression levels were also decreased after clodronate liposome treatment (Figure 3E). The number of spinal F4/80^+^CD11b^+^cells decreased after clodronate liposome treatment (Figure 3F,G). Hence, depletion of peripheral macrophage could suppress the spinal macrophage recruitment and inhibit spinal neuroinflammation to ameliorate pain in chronic prostatitis.

### 3.4 Blockade of CXCR3 Attenuates Pain Response and Spinal Macrophage Infiltration

**FIGURE 4:**
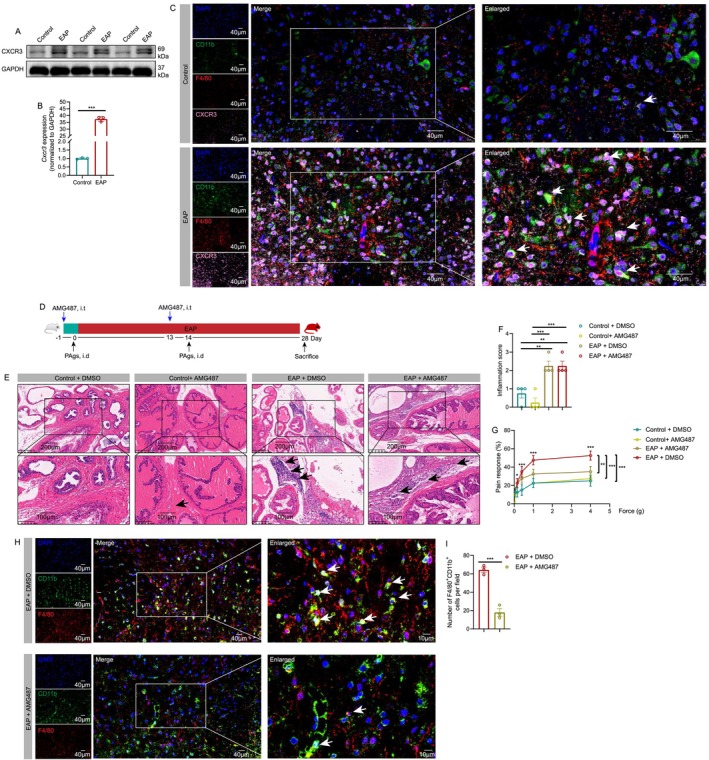
Suppression of CXCR3 attenuates pain response and macrophage infiltration in the spinal cord of EAP mice. Results of western blotting and qPCR showed the expression of CXCR3 protein levels and *Cxcr3* mRNA levels in the spinal cord of control and EAP mice (A and B). The results of the immunofluorescence assay displayed the expression levels of CXCR3 in F4/80^+^CD11b^+^ macrophages in the spinal cord of control and EAP mice (C). Results of H&E staining showed the effects of intrathecal injection of AMG487 on the prostate inflammation of EAP mice (D–F). Effects of AMG487 on pain response in EAP mice (G). Effects of AMG487 treatment on macrophage infiltration in the spinal cord of EAP mice (H and I). EAP: experimental autoimmune prostatitis; i.t.: intrathecally. *p < 0.05; ***p < 0.001.

CXCL10 was a ligand of CXCR3, which might bind to CXCR3 to attract macrophage infiltration in the spinal cord to induce neuroinflammation and chronic prostatitis pain symptoms. Results of western blotting and qPCR showed that the expression levels of CXCR3 protein and *Cxcr3* mRNA were higher in the spinal cord of EAP mice (Figure 4A,B). Immunofluorescence assay found that CXCR3 was elevated in spinal F4/80^+^CD11b^+^ macrophages (Figure 4C). Based on the vital roles of the CXCL10/CXCR3 axis in macrophage attraction and immune responses, we used AMG487, an antagonist of CXCR3, to intrathecally inject into EAP mice. Results of H&E staining showed that intrathecal injection of AMG487 had no effects on the prostate inflammation of EAP mice (Figure 4D–F). However, administration of AMG487 reduced pain response in EAP mice compared to EAP mice treated with DMSO (Figure 4G). Moreover, AMG487 treatment decreased spinal macrophage infiltration (Figure 4H,I). Therefore, the CXCL10/CXCR3 axis enhanced the recruitment of spinal macrophages, and blockade of CXCR3 could reduce macrophage infiltration to attenuate pain response in EAP mice.

### 3.5 The Altered Proteome in Spinal Macrophages Between Control and EAP Mice

**FIGURE 5:**
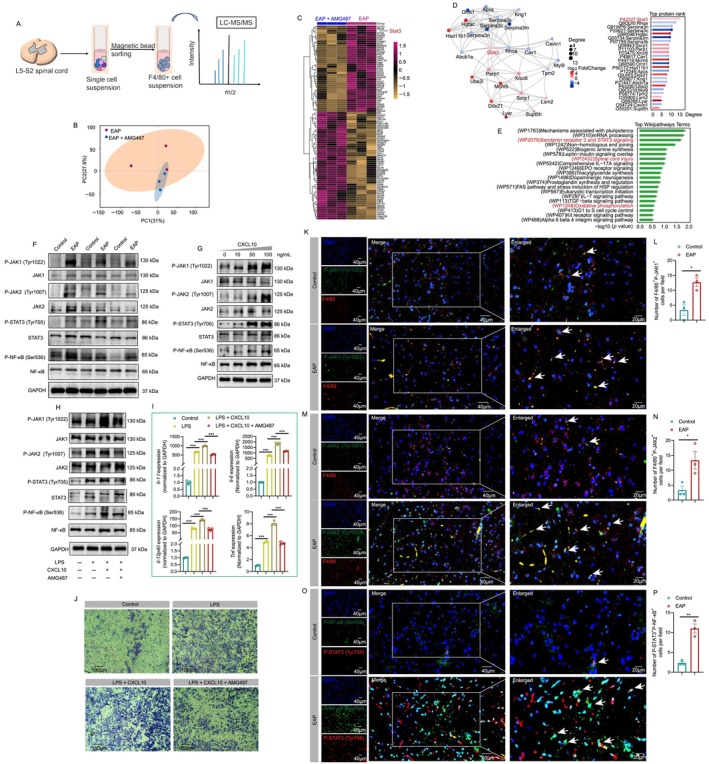
The JAK/STAT3 pathway is involved in the cytokine secretion and migration of spinal macrophages. The whole process of isolation of spinal macrophages (A). Principal component analysis of the proteomic results (B). The heatmap showed the differentially expressed proteins between the spinal macrophages in EAP and EAP + AMG487 groups (C). The interaction among these differentially expressed proteins (D). The functional annotation results based on the differentially expressed proteins (E). The protein levels of the P‐JAK1, JAK1, P‐JAK2, JAK2, P‐STAT3, STAT3, P‐NF‐κB and NF‐κB in the spinal cord of EAP and control groups (F). The expression levels of P‐JAK1, JAK1, P‐JAK2, JAK2, P‐STAT3, STAT3, P‐NF‐κB and NF‐κB in macrophages with CXCL10 treatment (G). The expression levels of P‐JAK1, JAK1, P‐JAK2, JAK2, P‐STAT3, STAT3, P‐NF‐κB and NF‐κB in control, LPS, LPS + CXCL10 and LPS + CXCL10 + AMG487 groups (H). Results of qPCR showed the mRNA expression levels of *Il‐1β*, *Il‐6*, *Il‐12p4*0 and *Tnf* in control, LPS, LPS + CXCL10 and LPS + CXCL10 + AMG487 groups (I). The effects of LPS, CXCL10 and AMG487 on the migration of RAW264.7 cells (J). Results of immunofluorescence showed the expressions of P‐JAK1 and P‐JAK2 in spinal macrophages in EAP and control mice (K–N). Results of immunofluorescence showed the expressions of P‐STAT3 and P‐NF‐κB in the spinal cord of EAP and control mice (O–P). EAP: experimental autoimmune prostatitis. *p < 0.05; **p < 0.01; *** *p* < 0.001.

Because macrophages elicited an important effect on the development of inflammatory diseases, and the spinal RNA sequence also identified that elevated CXCL10 levels are associated with spinal macrophage infiltration and neuroinflammation, we isolated F4/80^+^ cells from the spinal cords of EAP and EAP + AMG487 mice to perform proteome analysis. Figures 5A and S5A showed the results of principal component analysis and the differentially expressed proteins between EAP and control mice by using a volcano plot. The heatmap further showed that among these differentially expressed proteins, STAT3 was elevated in the spinal macrophages of EAP mice (Figure 5C). All differentially expressed proteins can be found in Table S5. The PPI network showed the association among these differentially expressed proteins (Figure 5D). Moreover, function annotation found that these differentially expressed proteins were associated with ‘Spinal cord injury’, ‘Serotonin receptor 2 and STAT3 signaling’, ‘MET activates STAT3’, ‘PTK6 activates STAT3’ and ‘Oxidative phosphorylation’ (Figures 5E and S5B–D). As the signal transducers and activator of transcription (STAT) family member, STAT3 was decreased in the spinal macrophages after suppressing CXCR3 in our proteomic analysis. Janus kinase (JAK) could activate STAT3, and the activated STAT3 (phosphorylation of STAT3, P‐STAT3) was associated with the secretion of immune mediators in inflammatory responses [44]. Hence, the JAK/STAT3 pathway in spinal macrophage may play an important role in the CXCL10/CXCR3 axis‐induced neuroinflammation and mediate pain response in chronic prostatitis.

### 3.6 The JAK/STAT3 Pathway Is Downstream of CXCL10/CXCR3 to Mediate Cytokines Secretion and Migration of Macrophage

A previous study reported that STAT3 and NF‐κB could work together to regulate the functions of macrophages [45], hence, the levels of the spinal JAK/STAT3 pathway and NF‐κB in EAP mice were detected. The in vitro experiments were performed to explore the roles of the CXCL10/CXCR3 axis in regulating the JAK/STAT3 pathway to attract macrophages and induce cytokine secretion. The protein levels of spinal P‐JAK1, P‐JAK2, P‐STAT3 and P‐NF‐κB were higher in EAP mice than in the control mice (Figure 5F). Results showed that with the elevation in CXCL10 concentration, the protein levels of P‐JAK1, P‐JAK2, P‐STAT3 and P‐NF‐κB were increased (Figure 5G). Moreover, LPS and LPS + CXCL10 increased P‐JAK1, P‐JAK2, P‐NF‐κB and P‐STAT3 protein levels, while AMG487 decreased the expressions of these proteins (Figure 5H). Results of qPCR showed that LPS and LPS + CXCL10 increased and AMG487 decreased the mRNA expression levels of *Il‐1β*, *Il‐6*, *Il‐12p4*0 and *Tnf*, respectively (Figure 5I). In addition, the migration assay showed that LPS and LPS + CXCL10 promoted the migration of RAW264.7 cells, while AMG487 inhibited the migration (Figure 5J). Immunofluorescence showed that the P‐JAK1 and P‐JAK2 were co‐localised with spinal macrophages in EAP mice, indicating that P‐JAK1 and P‐JAK2 were expressed in spinal macrophages, and compared to control mice, the levels of P‐JAK1 and P‐JAK2 were higher (Figure 5K–N). Moreover, P‐STAT3 was found to co‐localise with P‐NF‐κB in the spinal cord of EAP mice (Figure 5O,P). From the above mentioned results, we could find that CXCL10 promoted the migration and cytokine secretions of macrophages through the JAK/STAT3 pathway, which may exert a lot in the occurrence of neuroinflammation and pain in chronic prostatitis.

### 3.7 Blockade of P‐STAT3 Inhibits the Migration and Cytokine Secretion of Macrophages

**FIGURE 6:**
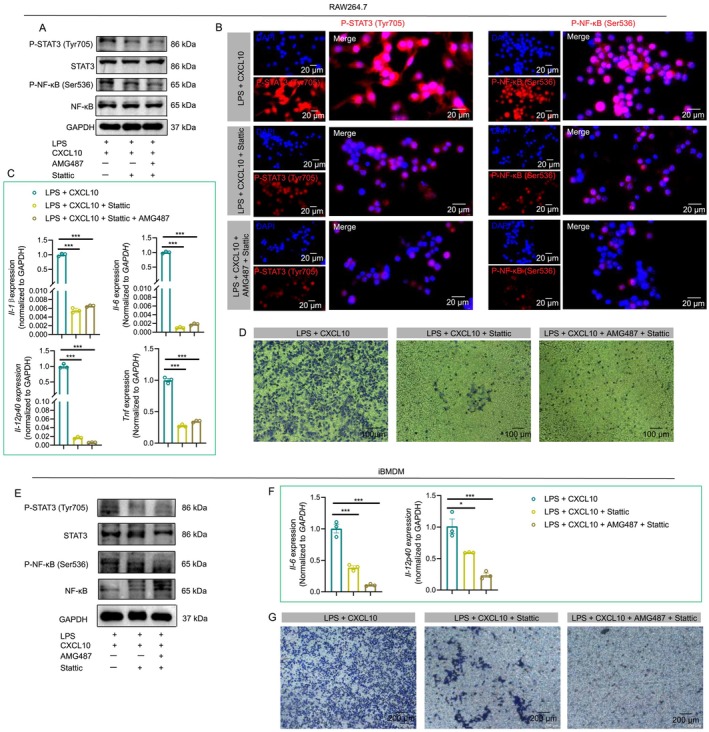
Blockade of P‐STAT3 inhibits the migration and cytokine secretion of macrophages. The expression levels of P‐STAT3, STAT3, P‐NF‐κB, and NF‐κB in LPS + CXCL10, LPS + CXCL10 + stattic, and LPS + CXCL10 + stattic + AMG487 groups in RAW264.7 cells (A‐B). The mRNA expression levels of *Il‐1β*, *Il‐6*, *Il‐12p4*0 and *Tnf* in cells treated with LPS + CXCL10, LPS + CXCL10 + stattic and LPS + CXCL10 + stattic + AMG487 (C). The effects of stattic on the migration of macrophages (D). In iBMDM cells, the expression levels of P‐STAT3, STAT3, P‐NF‐κB and NF‐κB in LPS + CXCL10, LPS + CXCL10 + stattic and LPS + CXCL10 + stattic + AMG487 groups (E). The mRNA expression levels of *Il‐6 and Il‐12p4*0 in cells treated with LPS + CXCL10, LPS + CXCL10 + stattic and LPS + CXCL10 + stattic + AMG487 (F) and the effects of stattic on the migration of macrophages (G). *p < 0.05; ***p < 0.001.

We further explored the effects of P‐STAT3 inhibition on macrophage migration and P‐NF‐κB pathway activation. In RAW264.7 cells, blockade of P‐STAT3 with stattic suppressed P‐STAT3 and P‐NF‐κB expression levels (Figure 6A), and stattic + AMG487 treatment further decreased P‐STAT3 and P‐NF‐κB expression. Moreover, the immunofluorescent intensities of P‐STAT3 and P‐NF‐κB were also decreased after stattic treatment in RAW264.7 cells (Figure 6B). Stattic and AMG487 inhibited the expression levels of *Il‐1β*, *Il‐6*, *Il‐12p4*0 and *Tnf* (Figure 6C). Finally, the migration abilities of macrophages were also inhibited in response to stattic and stattic + AMG487 treatment (Figure 6D). In iBMDM cells, blockade of P‐STAT3 and CXCR3 decreased P‐STAT3 and P‐NF‐κB expression (Figure 6E). The expression levels of *Il‐6* and *Il‐12p4*0 were inhibited in response to stattic and AMG487 treatment (Figure 6F), and the migration abilities of macrophages were also inhibited (Figure 6G). Taken together, the CXCL10/CXCR3 axis could regulate the migration and cytokine secretion of macrophages via the JAK/STAT3 and NF‐κB pathways.

### 3.8 The Prostate NGF/TrKA Axis Could Induce Neuroinflammation to Mediate Pain Response in Chronic Prostatitis

**FIGURE 7:**
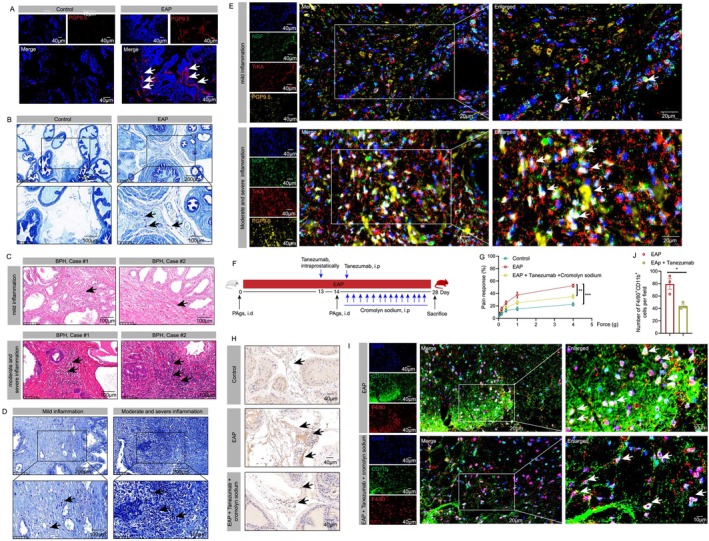
The prostate NGF/TrKA axis induces neuroinflammation to mediate pain response in chronic prostatitis. The expression levels of PGP9.5 in the prostates of EAP and control mice (A). The number of mast cells in the prostates of EAP and control mice (B). The number of mast cells in BPH patients with mild, moderate and severe prostate inflammation (C and D). The expression levels of NGF, TrKA and PGP9.5, and the co‐localization relationship among them in BPH patients' prostate tissues with mild, moderate and severe prostate inflammation (E). The effects of tanezumab and cromolyn sodium on pain responses in EAP mice (F and G), and the expression levels of NGF in the prostates of EAP mice treated with tanezumab and cromolyn sodium (H). The number of infiltrated macrophages in the spinal cord of EAP mice treated with tanezumab and cromolyn sodium (I and J). BPH: benign prostate hyperplasia; EAP: experimental autoimmune prostatitis; NGF: nerve growth factor. *p < 0.05; ***P < 0.01; ***P < 0.001.

Previous studies reported that several local immune mediators including NGF could bind to sensory neurons to mediate pain, hence, we explored the roles of the NGF/TrKA axis in pain responses in chronic prostatitis. We found that compared to control mice, PGP9.5 was highly expressed in the prostates of EAP mice, indicating that the densities of nerve fibres were increased (Figure 7A). As an important source of NGF, mast cells were increased in the prostates of EAP mice (Figure 7B). Moreover, compared to patients with mild prostate inflammation, the amount of mast cells was also increased in BPH patients with moderate and severe prostate inflammation (Figure 7C,D). The expression levels of NGF, TrKA, PGP9.5 and the co‐localisation relationship among them were detected in BPH patients' prostate tissues, and results showed that NGF, TrKA and PGP9.5 were highly expressed in patients' prostates with moderate and severe prostate inflammation, and NGF, TrKA and PGP9.5 were also co‐localised in the prostates of BPH patients (Figure 7E). Hence, the NGF may bind to the TrKA to activate the sensory neurons to mediate neuroinflammation and pain response in chronic prostatitis.

It is reported that targeting NGF (tanezumab) and stabilisation of mast cells (cromolyn sodium) could partially suppress pain response in EAP mice [27], we applied tanezumab and cromolyn sodium to EAP mice, and we found that inhibition of NGF and stabilisation of mast cells could reduce pain responses in EAP mice (Figure 7F,G). The levels of NGF in the prostates of EAP mice decreased after tanezumab and cromolyn sodium administration (Figure 7H). The infiltrated spinal macrophages in EAP mice treated with tanezumab and cromolyn sodium were decreased (Figure 7I,J). Taken together, the abovementioned results showed that local NGF in the prostate was involved in the occurrence of pain in chronic prostatitis and NGF was a potential target in pain management in chronic prostatitis.

## 4 Discussion

**FIGURE 8:**
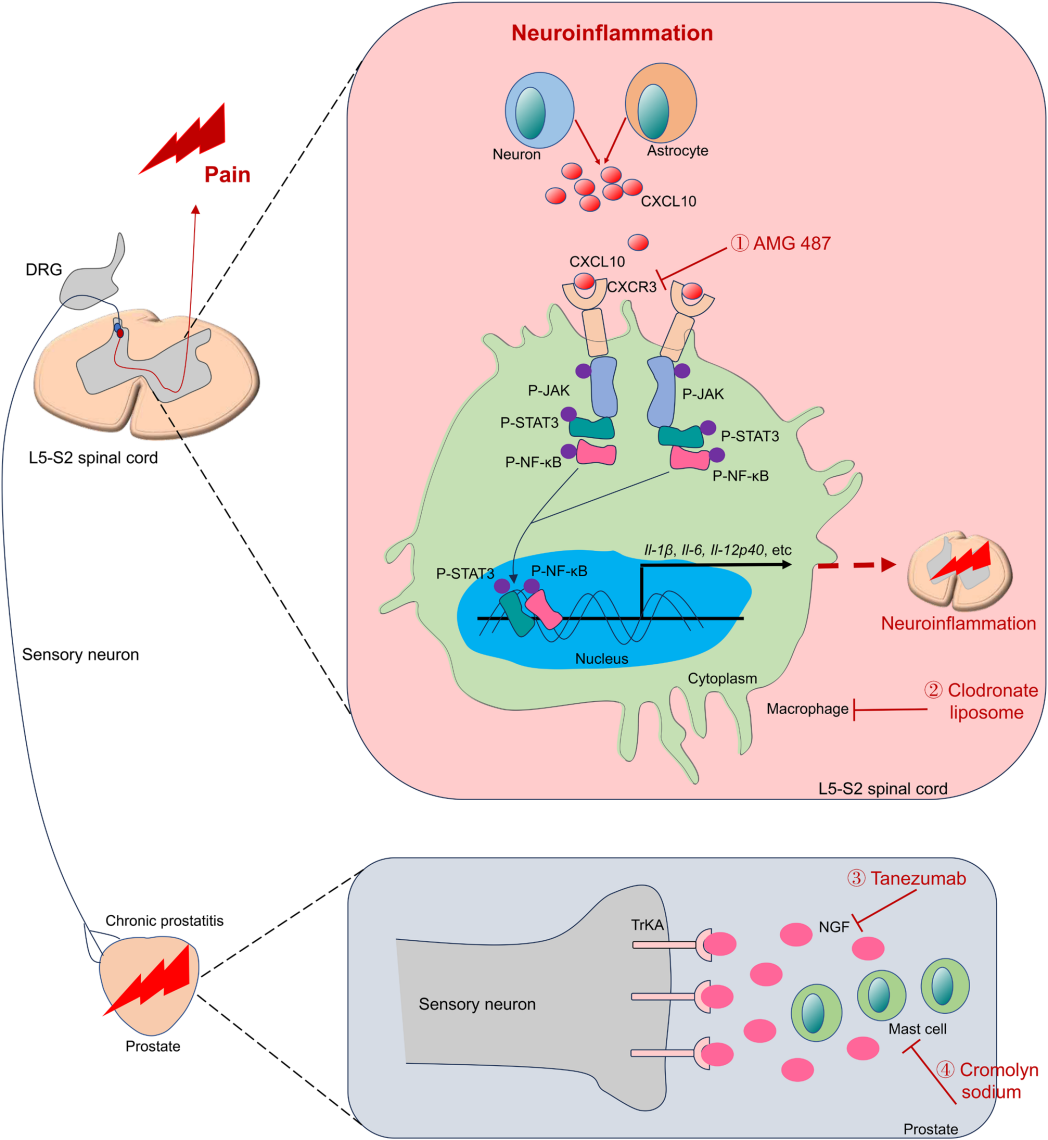
The schematic illustration of the NGF/TrKA, CXCL10/CXCR3 and JAK/STAT3 pathways in the initiation of pain in chronic prostatitis. In the prostate of EAP mice, the mast cell‐derived NGF could bind the TrKA in the sensory neurons, which indued the activated spinal neurons and astrocytes to release CXCL10. CXCL10 bound with CXCR3 to stimulate the JAK/STAT3 pathway and the NF‐κB pathway, and the spinal macrophages released the pro‐inflammatory cytokines to induce neuroinflammation. The CXCR3 inhibitor (AMG487), an NGF antibody (tanezumab), a mast cell stabiliser (cromolyn sodium) and macrophage depletion (clodronate liposome) show the potential for chronic prostatitis treatment. EAP: experimental autoimmune prostatitis; NGF: nerve growth factor.

In this study, we explored the roles of the CXCL10/CXCR3 axis in regulating spinal macrophage recruitment and cytokine secretion to exacerbate neuroinflammation and pain in chronic prostatitis, and our main findings were as follows: (1) spinal macrophages and pro‐inflammatory cytokine levels were increased in EAP mice, and depletion of macrophage attenuated pain response; (2) neuron and astrocyte‐derived CXCL10 could attract macrophages to the spinal cord and inducing neuroinflammation, and blockade of the CXCL10/CXCR3 axis decreased spinal macrophages infiltration and pain response in EAP mice; (3) the JAK/STAT3 pathway was downstream of the CXCL10/CXCR3 axis to induce the migration and cytokine secretion of spinal macrophages; (4) the prostate NGF/TrKA axis was involved in the spinal macrophages recruitment and pain response, and targeting NGF was effective in pain treatment in chronic prostatitis. Taken together, these findings demonstrated the important roles of the CXCL10/CXCR3 axis in modulating the JAK/STAT3 pathway and inducing neuroinflammation to mediate pain in chronic prostatitis, and targeting the NGF/TrKA pathway showed therapeutic efficacy in pain treatment in chronic prostatitis (Figure 8).

Chronic prostatitis is one of the most common urologic diseases in young men, symptoms including pelvic pain, sexual dysfunction and voiding puzzle most chronic prostatitis patients, which severely reduces the quality of life [1]. For patients with long‐term chronic prostatitis, depression may occur, which in turn aggravates pelvic pain [3, 46]. The mechanisms underlying pain in chronic prostatitis have not been fully elucidated. The prostate was innervated by the L5‐S2 spinal cord, and the activated microglia, cytokines and chemokines including TNF‐α, CCL3 and IL‐1β were increased in the spinal cord of the EAP mice [42, 47]. Moreover, activated astrocytes and iNOS levels were increased in the spinal cord of EAP mice [48], and electroacupuncture could inhibit the activated astrocytes to attenuate pain in chronic prostatitis [49]. EAP induction could attract peripheral monocytes and macrophages to the central nervous system. CCL2 was increased in the prostate and spinal cord of the EAP model, and CCL2 was associated with prostate, DRG and spinal cord macrophage infiltration [23]. The Notch was found to promote microglia activation and cytokine release to induce pain in chronic prostatitis, and inhibition of spinal macrophages and microglia could improve the tactile allodynia in the EAP model [10, 23]. Hence, microglia, macrophages and astrocytes were associated with pain in chronic prostatitis [9]. However, mechanisms underlying spinal macrophage infiltration and activation were not fully elucidated, and more studies were needed to find novel therapeutic targets for pain management.

In this study, we found that the expression levels of spinal pro‐inflammatory mediators and spinal macrophage infiltration were increased in EAP mice, which was in line with the previous study [23, 48]. Depletion of macrophage could effectively attenuate spinal macrophage recruitment and pain response in EAP mice, indicating the important roles of macrophage in neuroinflammation and pain development in chronic prostatitis. Subsequently, the RNA sequence found that CXCL10 and CCR2 were highly expressed in the SCDH of L5‐S2. Because the roles of the CCL2/CCR2 axis in spinal macrophage recruitment have been elucidated [23], we focus on the roles of CXCL10 in neuroinflammation and pain in chronic prostatitis. The results demonstrated that spinal CXCL10 expression levels were increased in EAP mice, and neurons and astrocytes were potential sources of spinal CXCL10. Because CXCR3 was the receptor of CXCL10, we assumed that CXCL10 may function through CXCR3. We found that spinal CXCR3 expression levels were increased in EAP mice, and CXCL10 and CXCR3 were co‐localised in spinal macrophages. To further explore the roles of CXCR3 in spinal macrophage recruitment and pain in EAP mice, CXCR3 inhibitor AMG487 was administrated to EAP mice, and blockade of CXCR3 reduced the number of infiltrated spinal macrophages and pain responses, which demonstrated that the CXCL10/CXCR3 axis was involved in spinal macrophages recruitment and pain in chronic prostatitis.

Chen et al. reported that the spinal and DRG expression levels of CXCL10 and CXCR3 were increased in chronic constriction injury, and inhibition of CXCR3 attenuated mechanical allodynia in rats, and p‐ERK was identified as a downstream of the CXCL10/CXCR3 axis [21, 22]. Our previous study showed that the CXCL10/CXCR3 axis could activate the p38 and ERK to induce macrophage migration to the prostate and secret cytokines [24]. Hence, p38 and ERK are the important downstream of the CXCL10/CXCR3 axis. For tissue‐resident macrophages, Zhang et al. reported that the JAK/STAT1 pathway was downstream of the CXCL10/CXCR3 axis to induce M1 macrophage polarisation to exacerbate liver fibrosis [30]. However, the relationship between the CXCL10/CXCR3 axis and the JAK/STAT3 pathway in neuroinflammation and pain in chronic prostatitis was unknown.

To investigate the mechanisms underlying the CXCL10/CXCR3‐induced neuroinflammation, EAP mice were intrathecally injected with a CXCR3 antagonist, and the proteome analysis found that the levels of STAT3 in spinal macrophages were decreased. Because STAT3 was regulated by the JAK, we proposed that the JAK/STAT3 pathway may be downstream of the CXCL10/CXCR3 axis to induce spinal macrophage recruitment and pain in chronic prostatitis. Our results also showed that the expression levels of P‐JAK1 and P‐JAK2 were elevated in spinal macrophages of EAP mice. STAT3 was reported to affect the activity of NF‐κB, and NF‐κB could also modulate the activity of STAT3 [45]. Hence, we proposed that the NF‐κB pathway may be also engaged in spinal macrophage recruitment and neuroinflammation through interacting with STAT3. Our results demonstrated that P‐STAT3 and P‐NF‐κB were increased and co‐localised in the spinal cord of EAP mice. In addition, CXCL10 could activate the JAK/STAT3 and NF‐κB pathways, and the expression levels of pro‐inflammatory cytokines were also increased in response to CXCL10 stimulation, while blockade of CXCR3 showed the reverse effects. Macrophage migration assay also found that CXCL10 enhanced macrophage migration while the CXCR3 antagonist suppressed the migration. The stattic significantly inhibited CXCL10‐induced P‐STAT3 and P‐NF‐κB expressions and macrophage migration. Taken together, the abovementioned results demonstrated that neuron and astrocyte‐derived CXCL10 could recruit and activate the spinal macrophages to induce spinal neuroinflammation and pain through the JAK/STAT3 pathway.

NGF was involved in neurons and pain development [50, 51]. NGF was elevated in pain and inflammatory diseases including arthritis, and NGF was associated with neuroinflammation and inflammatory pain development [52, 53, 54]. In addition, NGF and its receptor TrKA have been demonstrated to be involved in maintaining inflammatory and neuropathic pain [55, 56, 57]. Monoclonal antibodies and small molecules targeting the NGF/TrKA pathway have been investigated a lot, which was warranted for pain treatment [57, 58, 59, 60]. For example, an NGF antibody tanezumab was applied to treat painful osteoarthritis, and administration of tanezumab could improve the pain symptoms of patients [26, 61, 62]. Done et al. applied tanezumab to EAP mice and pain reduction was not significant, and not sustained tanezumab treatment or method of tanezumab administration were possible reasons for these insignificant results [27]. Mast cells and NGF were also elevated in EAP mice, and the elimination of mast cells was demonstrated to reduce pain in EAP mice [27]. Done et al. also displayed that a mast cell stabiliser cromolyn sodium could partially attenuate pain response in EAP mice. Because mast cell and NGF have important roles in pain in chronic prostatitis, cromolyn sodium and tanezumab (ip and intra‐prostatic injection) were simultaneously administrated to EAP mice, and the results showed that cromolyn sodium and tanezumab could reduce pain in EAP mice, and the spinal macrophages were also decreased, showing that targeting mast cell and NGF was warranted for chronic prostatitis, and more exploration should be conducted to demonstrate their combined efficacies in pain management.

## 5 Conclusion

In conclusion, the CXCL10/CXCR3 axis was involved in spinal macrophage infiltration through activating the JAK/STAT3 pathway. The prostate NGF/TrKA axis was involved in the neuroinflammation and pain in chronic prostatitis, and inhibition of NGF could attenuate pain response in chronic prostatitis, which provided novel therapeutic targets for pain treatment in chronic prostatitis.

## Author Contributions

**Lei Chen, Ziqi Chen, Jia Chen:** writing – original draft, data collection, methodology, data curation, formal analysis. **Hexi Du, Xianguo Chen:** methodology, data curation. **Jing Chen, Hui Wang, Chaozhao Liang:** writing – review and editing, funding acquisition, conceptualisation, supervision.

## Ethics Statement

All procedures followed were in accordance with the ethical standards of the Human Ethics Committee of FAHAMU and with the Helsinki Declaration.

## Consent

Informed consent was obtained from all patients in the study.

## Conflicts of Interest

The authors declare no conflicts of interest.

## Supporting information


Data S1.

## Data Availability

The data that support the findings of this study are available from the corresponding author upon reasonable request.
