# Supplementary material for: CXCL10 Promotes Spinal Macrophage Recruitment via the JAK/STAT3 Pathway to Induce Pain in Experimental Autoimmune Prostatitis
Source: Cell Prolif. 2024 Dec 24;58(4):e13784. doi: 10.1111/cpr.13784 (PMC11969258; doi:10.1111/cpr.13784)
Supplement: Supplementary file 1 — Data S1. [file CPR-58-e13784-s001.docx]

**CXCL10 promotes spinal macrophage recruitment via the JAK/STAT3 pathway to induce pain in experimental autoimmune prostatitis**

Lei Chen^1,2,3#^, Ziqi Chen^1,2,3#^, Jia Chen^1,2,3#^, Hexi Du^1,2,3^, Xianguo Chen^1,2,3^, Jing Chen^1,2,3*^, Hui Wang^1,2,3*^, Chaozhao Liang^1,2,3*^

^1^Department of Urology, the First Affiliated Hospital of Anhui Medical University

^2^Institute of Urology, Anhui Medical University

^3^Anhui Province Key Laboratory of Urological and Andrological Diseases Research and Medical Transformation, No. 218, Jixi Road, Hefei 230022, Anhui, China.

^#^ These authors contributed equally to this work.

*Correspondence: Jing Chen (ayd_chenjing@163.com), Hui Wang (whayd@sina.cn), Chaozhao Liang ([liang_chaozhao@ahmu.edu.cn](mailto:liang_chaozhao@ahmu.edu.cn))

Tel.: + 86 55162923932, Fax: + 86 55163633742

**Address:** No. 218, Jixi Road, Hefei 230022, Anhui, China.

Running title: CXCL10 Promotes Chronic Prostatitis Pain

**Word Count:** 6,133

**Figure number:** 8

**Supplemental Figure number:** 5

**Supplemental Table number:** 5

**Supplemental Figure 1. Results of RNA sequence analysis in the spinal cord of mice between the control and EAP mice.** The heatmap of the DEGs between the control and EAP mice (**A**). The function annotation results based on the Wikipathways database (**B**). The GSEA results of the identified genes (**C-D**). DEGs: differentially expressed genes; EAP: Experimental autoimmune prostatitis.

**
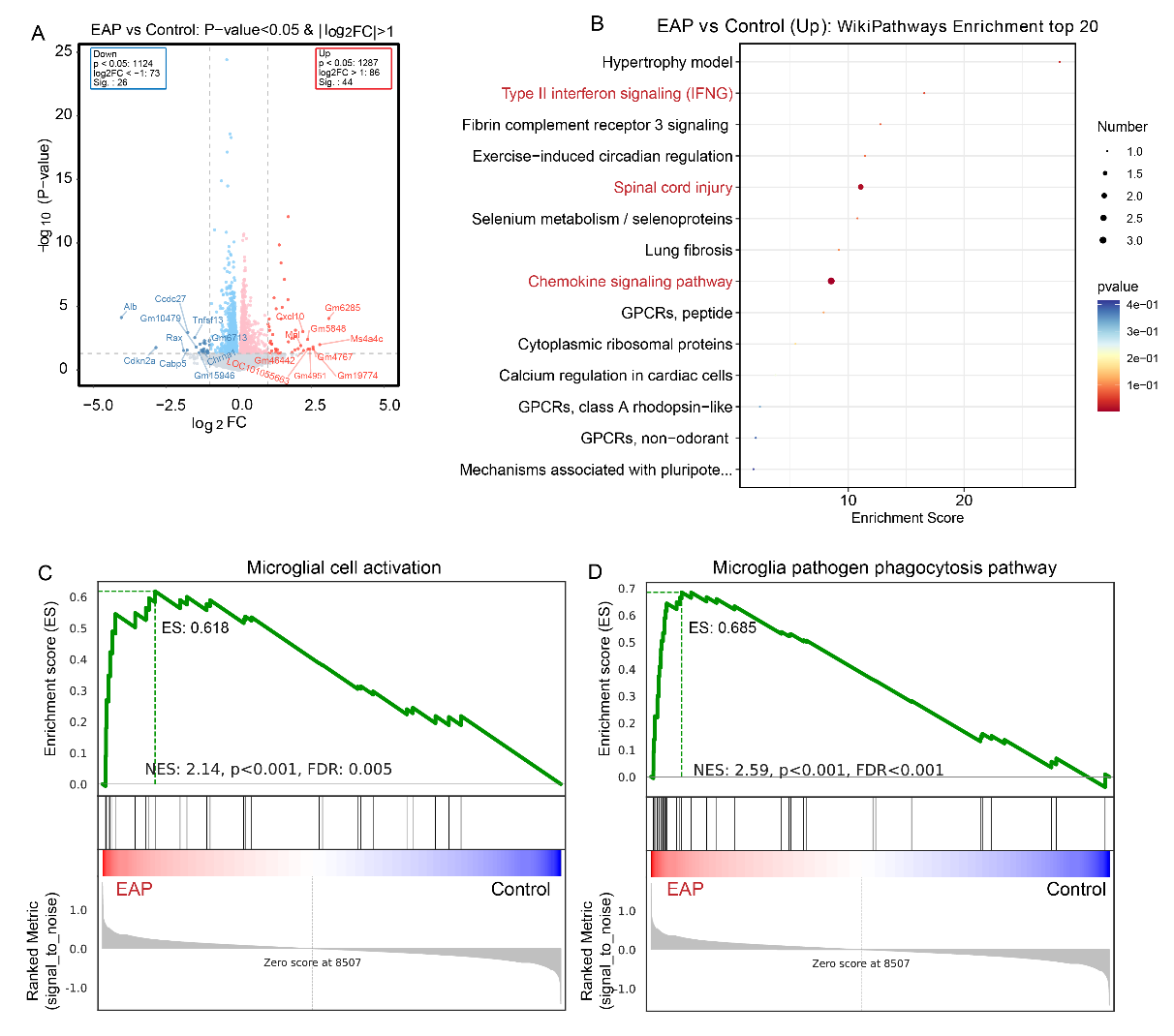
**

**Supplemental Figure 2. Function annotation of total DEGs.** The function annotation of total DEGs by using GO term (**A**), KEGG pathway (**B**), and Wikipathway (**C**). DEGs: differentially expressed genes.

**
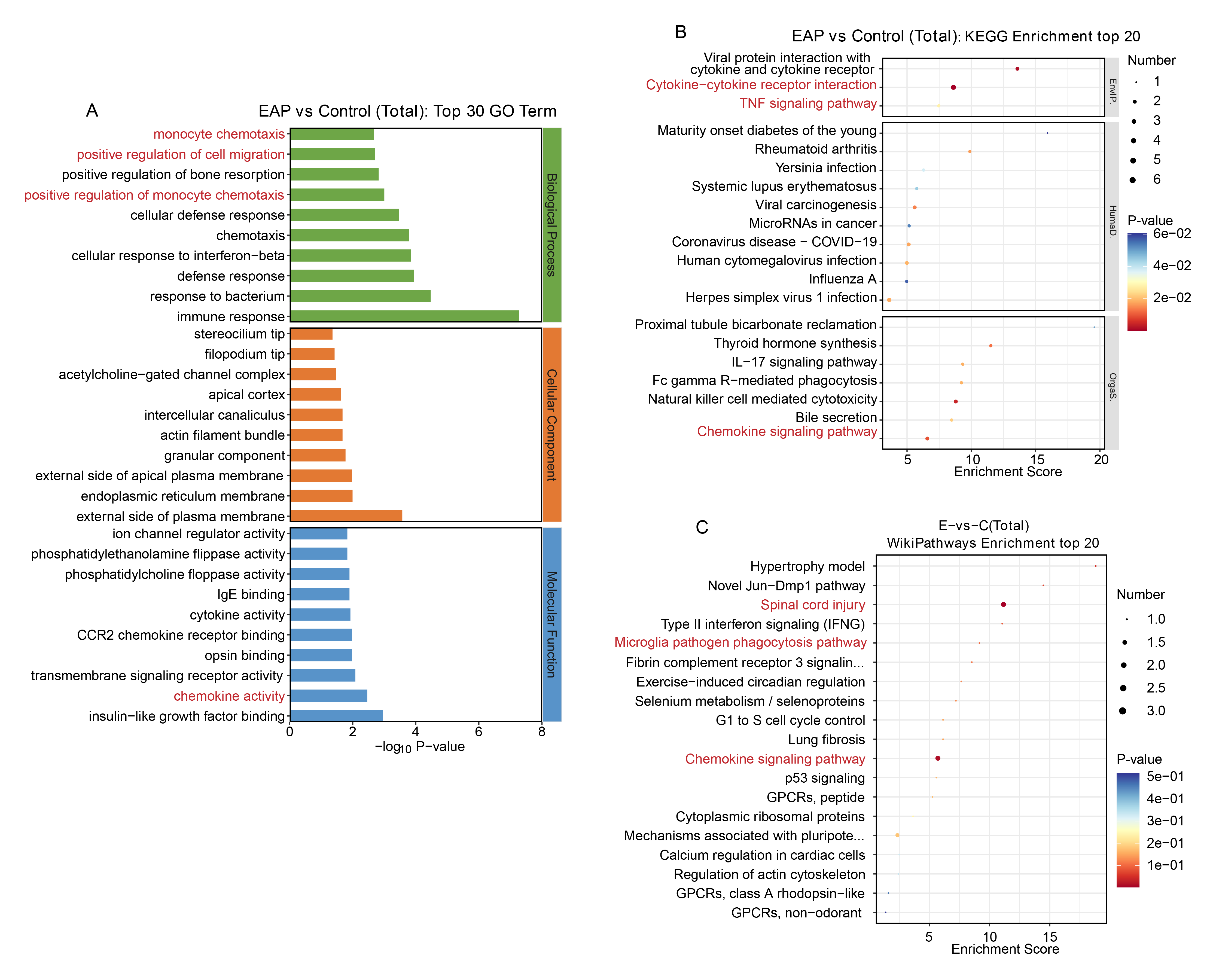
**

**Supplemental Figure 3. Function annotation of down-regulated DEGs.** The function annotation of down-regulated DEGs by using GO term (**A**), KEGG pathway (**B**), and Wikipathway (**C**). DEGs: differentially expressed genes.

**
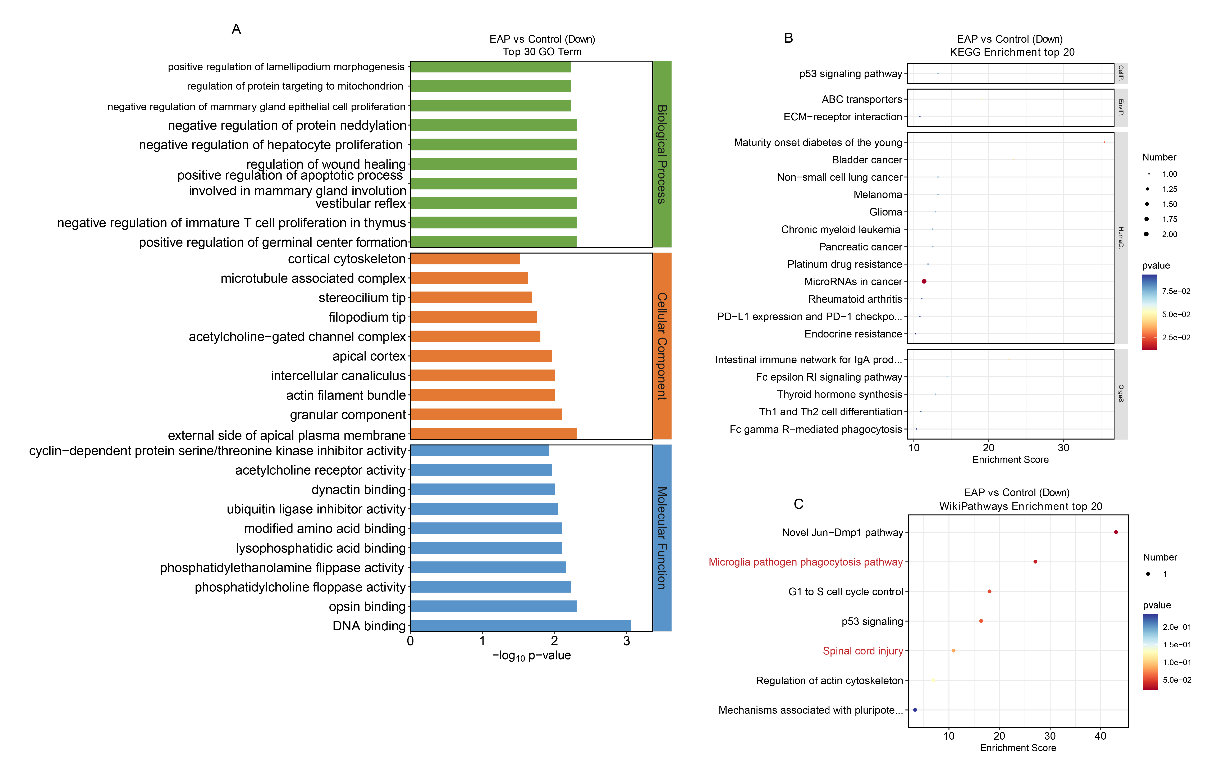
**

**Supplemental Figure 4. The alteration morphology of mice prostate at 0, 10, 20, and 28 days.** The whole process of EAP induction and treatment (**A**). Prostate inflammation at 0, 10, 20, and 28 days (**B-C**). The pain response of EAP mice at day 0, 10, 20, and 28 days (**D**). EAP: Experimental autoimmune prostatitis.

**
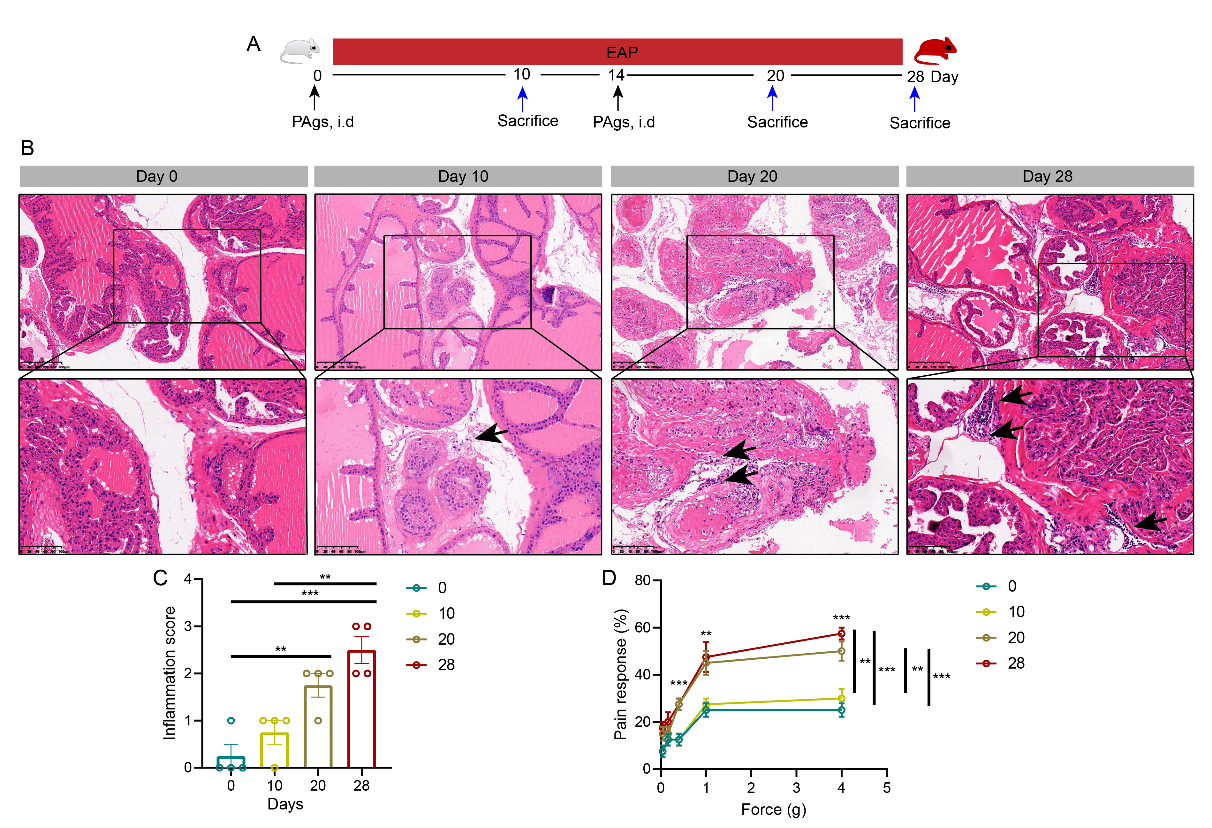
**

**Supplemental Figure 5. The altered proteome in spinal macrophages between control and EAP groups**. The differentially expressed proteins in the volcano plot between EAP and control mice (**A)**. The results of function annotation of these differentially expressed proteins (**B-D**). EAP: Experimental autoimmune prostatitis.


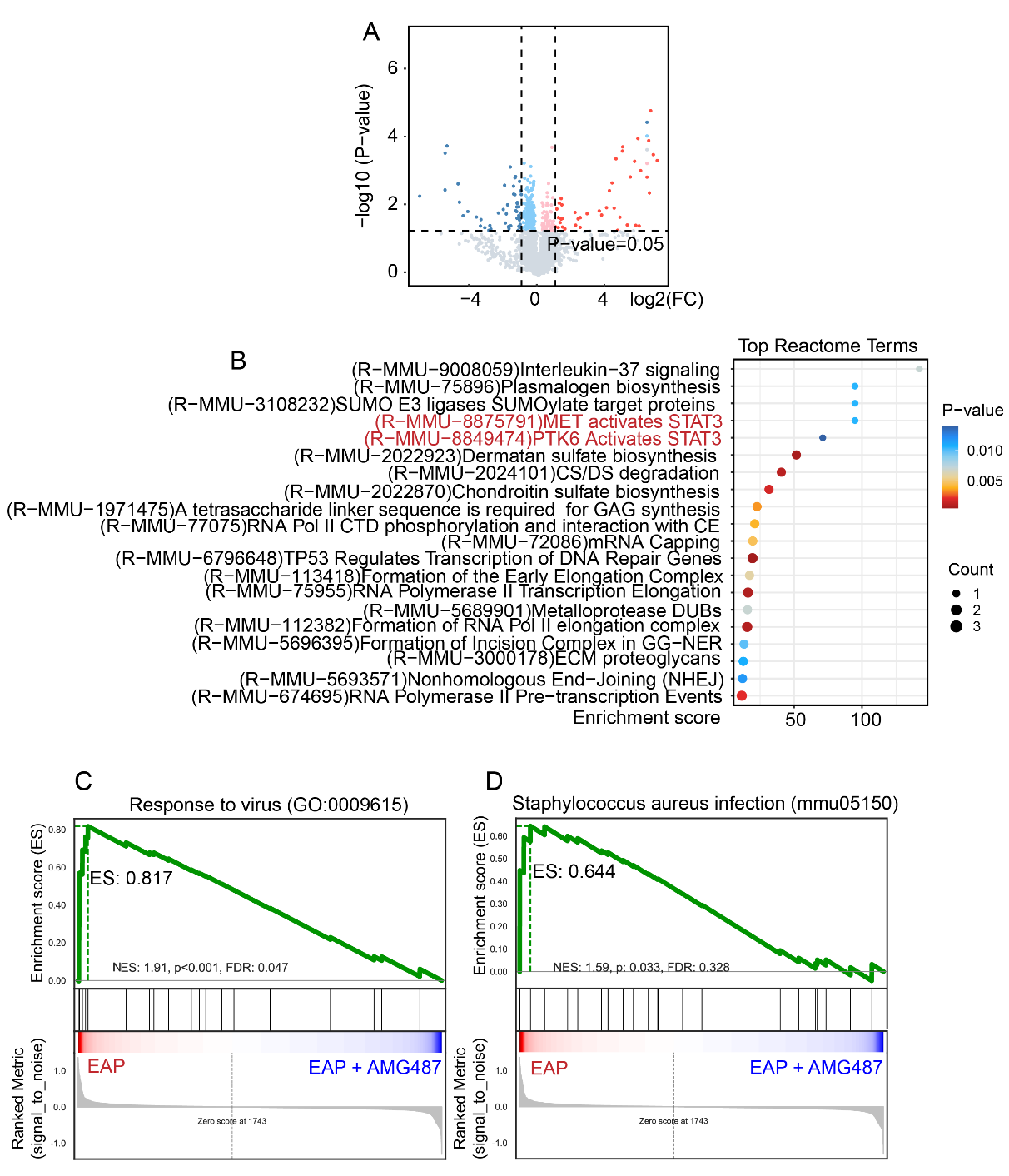


**Supplemental Table 1. Antibodies used in this study.**

|  | Cat | Company | Usage |
| --- | --- | --- | --- |
| P-JAK1 | AF2012 | Affinity | WB, IF |
| P-JAK2 | AF3024 | Affinity | WB, IF |
| P-NF-κB | AF2006 | Affinity | WB, IF |
| P-STAT3 | AF3293 | Affinity | WB, IF |
| F4/80 | GB113373-100 | Servicebio | IF |
| CD11b | DF2911 | Affinity | IF |
| CXCR3 | 26756-1-AP | Preteintec | WB, IF |
| CXCL10 | 10937-1-AP | Preteintec | WB, IF |
| NeuN | DF6145 | Affinity | IF |
| IBA-1 | DF6442 | Affinity | IF |
| GFAP | DF6040 | Affinity | IF |
| NGF | DF6061 | Affinity | IHC |
| TrKA | DF6822 | Affinity | IF |
| PGP9.5 | AF5490 | Affinity | IF |
| CD45 | DF6839 | Affinity | IF |
| JAK1 | AF5012 | Affinity | WB |
| JAK2 | AF6022 | Affinity | WB |
| NF-κB | AF5006 | Affinity | WB |
| STAT3 | AF6294 | Affinity | WB |
| GAPDH | AF7021 | Affinity | WB |
| Goat anti-rabbit | E-AB-1003 | Elabscience | WB |
| Rat Anti-Mouse F4/80 | 746070 | BD | FCM |
| Rat Anti-CD11b | 557396 | BD | FCM |

**Supplemental Table 2. Primers used in this study.**

| Genes | Forward | Reverse |
| --- | --- | --- |
| *Il-1β* | GCAACTGTTCCTGAACTCAACT | ATCTTTTGGGGTCCGTCAACT |
| *Il-6* | TAGTCCTTCCTACCCCAATTTCC | TTGGTCCTTAGCCACTCCTTC |
| *Il-12p40* | TGGTTTGCCATCGTTTTGCTG | ACAGGTGAGGTTCACTGTTTCT |
| *Tnf* | CCCTCACACTCAGATCATCTTCT | GCTACGACGTGGGCTACAG |
| *Gapdh* | AGGTCGGTGTGAACGGATTTG | TGTAGACCATGTAGTTGAGGTCA |

**Supplemental Table 3: Detailed information of the differentially expressed genes.**

| Gene symbol | Gene ID | Location | log2FoldChange | *P*-value | Up/down |
| --- | --- | --- | --- | --- | --- |
| Gm6285 | 622098 | ChrX: 104947755-104948329 | 3.098577 | 8.85E-05 | Up |
| Ms4a4c | 64380 | Chr19: 11404717-11427246 | 2.78661 | 0.01003 | Up |
| Gm19774 | 102632739 | Chr18: 38714323-38714765 | 2.561403 | 0.0221 | Up |
| Gm4767 | 210583 | Chr10: 81811937-81823898 | 2.554423 | 0.01496 | Up |
| Gm4951 | 240327 | Chr18: 60212004-60248440 | 2.444339 | 0.02203 | Up |
| LOC101055663 | 101055663 | Chr1: 68248-77556 | 2.382337 | 0.02302 | Up |
| Gm5848 | 545531 | Chr3: 68306190-68310535 | 2.370624 | 0.00392 | Up |
| Gm46442 | 108168140 | Chr14: 16451704-16452228 | 2.230064 | 0.02899 | Up |
| Cxcl10 | 15945 | Chr5: 92346638-92348889 | 2.208655 | 0.00095 | Up |
| Mpl | 17480 | Chr4: 118442412-118457688 | 2.140177 | 0.01203 | Up |
| Ccr2 | 12772 | Chr9: 124101918-124109140 | 2.047743 | 0.02079 | Up |
| H2bc15 | 319187 | Chr13: 21754076-21754553 | 1.958603 | 0.0023 | Up |
| Fam177a2 | 100101807 | Chr12: 55199264-55217168 | 1.952295 | 0.00074 | Up |
| Gm10224 | 100043805 | Chr6: 122698341-122699068 | 1.935965 | 0.0257 | Up |
| Gm20517 | 112694759 | Chr17: 47611585-47686739 | 1.836902 | 0.04122 | Up |
| Plac8 | 231507 | Chr5: 100551589-100572253 | 1.820116 | 0.00313 | Up |
| Dcstamp | 75766 | Chr15: 39745930-39760938 | 1.710503 | 0.00603 | Up |
| Gm5451 | 108168114 | Chr13: 66874062-66874753 | 1.703238 | 8.60E-13 | Up |
| Rybp-ps | 628746 | Chr13: 66007640-66012021 | 1.694239 | 2.89E-06 | Up |
| Gm6988 | 629595 | Chr12: 110315927-110316427 | 1.571442 | 7.40E-08 | Up |
| Tgtp2 | 100039796 | Chr11: 49057196-49064212 | 1.495432 | 1.16E-05 | Up |
| Ifi27l2a | 76933 | Chr12: 103433873-103443680 | 1.458244 | 9.20E-05 | Up |
| Gm7206 | 101056029 | Chr14: 45713346-45713826 | 1.455552 | 3.75E-09 | Up |
| Gm8909 | 667977 | Chr17: 36143812-36168602 | 1.440557 | 0.04553 | Up |
| Gm15501 | 100040298 | Chr7: 93178962-93184176 | 1.394507 | 1.43E-10 | Up |
| Gm7429 | 664969 | ChrX: 114980925-114981385 | 1.356215 | 0.04871 | Up |
| Fam177a | 73385 | Chr12: 55124435-55142104 | 1.338317 | 0.0249 | Up |
| Gm14438 | 101055915 | Chr2: 175261051-175271246 | 1.287408 | 1.52E-05 | Up |
| Fcgr4 | 246256 | Chr1: 171018926-171029761 | 1.281741 | 0.02563 | Up |
| Ccl12 | 20293 | Chr11: 82101845-82103399 | 1.279425 | 0.03922 | Up |
| Tnfrsf8 | 21941 | Chr4: 145267132-145315164 | 1.269291 | 0.02154 | Up |
| Gm10029 | 100039532 | Chr13: 6662535-6662972 | 1.216477 | 2.09E-06 | Up |
| Usp18 | 24110 | Chr6: 121245906-121270917 | 1.169271 | 0.00163 | Up |
| Crybg2 | 230806 | Chr4: 134060731-134092504 | 1.14015 | 0.0087 | Up |
| Gvin1 | 74558 | Chr7: 106156535-106215340 | 1.13519 | 0.04905 | Up |
| Igfbpl1 | 75426 | Chr4: 45809507-45826827 | 1.135182 | 0.00889 | Up |
| Gm3636 | 100041151 | Chr14: 6735683-6757601 | 1.132329 | 0.0314 | Up |
| Ifi44 | 99899 | Chr3: 151730922-151749910 | 1.09447 | 0.00521 | Up |
| Tgtp1 | 21822 | Chr11: 48985329-48992246 | 1.079965 | 0.00723 | Up |
| Fxyd2 | 11936 | Chr9: 45399709-45410278 | 1.063142 | 0.00076 | Up |
| Rpl17-ps8 | 102632403 | ChrX: 96484841-96485497 | 1.049159 | 9.46E-05 | Up |
| Rpl13-ps6 | 100040416 | Chr3: 58987123-58987860 | 1.046214 | 0.00039 | Up |
| Ccn1 | 16007 | Chr3: 145646971-145649985 | 1.038653 | 0.04894 | Up |
| Depp1 | 213393 | Chr6: 116650684-116652847 | 1.011719 | 0.00024 | Up |
| Abcb1b | 18669 | Chr5: 8798147-8866314 | -1.00338 | 0.0107 | Down |
| Parpbp | 75317 | Chr10: 88091398-88146969 | -1.00389 | 0.00437 | Down |
| Hmmr | 15366 | Chr11: 40701388-40733464 | -1.01402 | 0.00525 | Down |
| Tmc1 | 13409 | Chr19: 20783456-21037126 | -1.05248 | 0.04347 | Down |
| Lat | 16797 | Chr7: 126363827-126369705 | -1.06771 | 0.03322 | Down |
| Sptbn5 | 640524 | Chr2: 120041493-120088913 | -1.0833 | 0.02888 | Down |
| Nkx6-3 | 74561 | Chr8: 23153271-23158948 | -1.11891 | 0.0487 | Down |
| Onecut1 | 15379 | Chr9: 74861921-74889648 | -1.15032 | 0.0083 | Down |
| Zfp345 | 545471 | Chr2: 150470991..150485063 | -1.15403 | 0.0401 | Down |
| Smim38 | 100040049 | Chr7: 145205823-145208119 | -1.18134 | 0.00496 | Down |
| Tecta | 21683 | Chr9: 42329622-42401690 | -1.18997 | 0.0077 | Down |
| Fbxl13 | 320118 | Chr5: 21483847-21645698 | -1.19367 | 0.02127 | Down |
| Vil1 | 22349 | Chr1: 74409384-74435560 | -1.22072 | 0.04606 | Down |
| Gm34362 | 102637593 | Chr14: 45175038-45176705 | -1.22163 | 0.02636 | Down |
| Sebox | 18292 | Chr11: 78503513-78505081 | -1.22928 | 0.03614 | Down |
| Gm33532 | 102636475 | Chr8: 12472134-12476321 | -1.2616 | 0.03536 | Down |
| Chrna1 | 11435 | Chr2: 73563281-73580338 | -1.28537 | 0.0267 | Down |
| Gm6713 | 626858 | Chr10: 85694381-85710999 | -1.3318 | 0.00922 | Down |
| Gm15946 | 108168333 | Chr17: 12960963-12963213 | -1.35895 | 0.04356 | Down |
| Gm10479 | 108167321 | Chr12: 20415521-20462084 | -1.4638 | 0.01543 | Down |
| Tnfsf13 | 69583 | Chr11: 69682577-69685554 | -1.51442 | 0.00287 | Down |
| Ccdc27 | 381580 | Chr4: 154026644-154042745 | -1.75228 | 0.00109 | Down |
| Cabp5 | 29865 | Chr7: 13385960-13408887 | -1.77514 | 0.02681 | Down |
| Rax | 19434 | Chr18: 65934639-65939089 | -1.89631 | 0.02921 | Down |
| Cdkn2a | 12578 | Chr4: 89274473-89294619 | -2.84861 | 0.01708 | Down |
| Alb | 11657 | Chr5: 90460870-90476602 | -4.04142 | 7.4E-05 | Down |

**Supplemental Table 4. The genes in the yellow module of the WGCNA results.**

| Genes |
| --- |
| Arl4d |
| Arrdc2 |
| Atf3 |
| B2m |
| Bst2 |
| C1qa |
| C1qb |
| C1qc |
| Capn3 |
| Ccl12 |
| Ccl5 |
| Cd14 |
| Cd300c2 |
| Cd37 |
| Cd53 |
| Cd68 |
| Cd74 |
| Cdkn1a |
| Cebpd |
| Cks1b |
| Cox4i2 |
| Csf1r |
| Csf3r |
| Csrnp1 |
| Ctsh |
| Ctss |
| Cxcl10 |
| Cxcl16 |
| Cyba |
| Cyth4 |
| Ddit4 |
| Ddx58 |
| Depp1 |
| Dok1 |
| Dusp6 |
| Errfi1 |
| Fam177a2 |
| Fam83d |
| Fcer1g |
| Fcgr1 |
| Fcgr3 |
| Fermt3 |
| Fgd2 |
| Gbp3 |
| Gbp5 |
| Gbp7 |
| Gimap6 |
| Gna15 |
| Gngt2 |
| Gpr4 |
| Gpx7 |
| H2-Ab1 |
| H2-D1 |
| H2-K1 |
| H2-Q4 |
| H2-T23 |
| Havcr2 |
| Hck |
| Id1 |
| Ifi27l2a |
| Ifi35 |
| Ifi44 |
| Ifi47 |
| Ifit1 |
| Ifit3 |
| Igsf6 |
| Igtp |
| Iigp1 |
| Irf7 |
| Irf8 |
| Irf9 |
| Irgm2 |
| Isg15 |
| Itgam |
| Klf2 |
| Klf4 |
| Lag3 |
| Laptm5 |
| Lgals3bp |
| Ly86 |
| Lyn |
| Lyz2 |
| Mpeg1 |
| Ms4a6d |
| Myo1f |
| Ncf1 |
| Nckap1l |
| Nfkbia |
| Oas1a |
| Oasl2 |
| Oxld1 |
| P2ry6 |
| Pik3ap1 |
| Pld4 |
| Pmaip1 |
| Psmb8 |
| Psmb9 |
| Ptpn6 |
| Rasal3 |
| Rps27rt |
| Rtp4 |
| Saa3 |
| Sap18b |
| Sgk1 |
| Slfn2 |
| Smim3 |
| Spi1 |
| Sprr1a |
| Stat1 |
| Tap1 |
| Tcim |
| Tgfb1 |
| Thbd |
| Tmem119 |
| Tmem252 |
| Tmem52 |
| Tnfaip8l2 |
| Trem2 |
| Trim30a |
| Txnip |
| Tyrobp |
| Unc93b1 |
| Usp18 |
| Wdr89 |
| Zfp36 |

**Supplemental Table 5. Identified differentially expressed proteins in spinal macrophages.**

| Accession | Gene Name | log_2_FoldChange | Up/down | *P*-value |
| --- | --- | --- | --- | --- |
| Q61191 | Hcfc1 | -9.57938 | Down | 0.004750389 |
| Q03734 | Serpina3m | -5.50950333 | Down | 0.003126358 |
| Q8BGR2 | Lrrc8d | -5.50501 | Down | 0.000255479 |
| Q9CZJ2 | Hspa12b | -5.39447 | Down | 0.000158017 |
| Q8K352 | Sash3 | -4.73864 | Down | 0.002053171 |
| P29621 | Serpina3c | -4.66110333 | Down | 0.007151129 |
| Q6WVG3 | Kctd12 | -4.43952667 | Down | 0.017891378 |
| Q91X17 | Umod | -4.16533 | Down | 0.013474011 |
| Q60590 | Orm1 | -3.65747333 | Down | 0.019597425 |
| Q8VDS8 | Stx18 | -3.42292667 | Down | 0.036116007 |
| Q9ERF3 | Skic8 | -3.39219333 | Down | 0.023260682 |
| Q6ZQ82 | Arhgap26 | -3.16444667 | Down | 0.04044549 |
| O35927 | Ctnnd2 | -2.92634333 | Down | 0.0485242 |
| Q60575 | Kif1b | -2.86475333 | Down | 0.034876038 |
| Q3TUA9 | Pomk | -2.80904 | Down | 0.044079247 |
| Q61686 | Cbx5 | -2.36214 | Down | 0.014876993 |
| Q8C9H6 | Strip2 | -2.07313667 | Down | 0.011717109 |
| Q9D9G3 | Chic2 | -1.99141333 | Down | 0.002275113 |
| Q56A07 | Scn2b | -1.94306333 | Down | 0.029291106 |
| Q9JK23 | Psmg1 | -1.89195333 | Down | 0.015797475 |
| P12246 | Apcs | -1.81961333 | Down | 0.008561944 |
| O08581 | Kcnk1 | -1.77132333 | Down | 0.037778941 |
| Q8BYJ6 | Tbc1d4 | -1.73429 | Down | 0.043555282 |
| Q9CQ19 | Myl9 | -1.65829333 | Down | 0.000656051 |
| P43275 | H1-1 | -1.63362667 | Down | 0.035563004 |
| P55772 | Entpd1 | -1.47222 | Down | 0.002412313 |
| O54724 | Cavin1 | -1.45755 | Down | 0.004150628 |
| Q6PIU9 |  | -1.38722667 | Down | 0.004290475 |
| P01902 | H2-K1 | -1.38050333 | Down | 0.001364283 |
| P49817 | Cav1 | -1.34985 | Down | 0.001251916 |
| Q9WVD5 | Slc25a15 | -1.33476667 | Down | 0.012789446 |
| Q91WP6 | Serpina3n | -1.28337667 | Down | 0.013507296 |
| Q63918 | Cavin2 | -1.27531333 | Down | 0.009887373 |
| Q6A028 | Swap70 | -1.25602667 | Down | 0.021452207 |
| Q8C120 | Sh3rf3 | -1.24930667 | Down | 0.007278727 |
| P21447 | Abcb1a | -1.24835333 | Down | 0.007778987 |
| Q9CVD2 | Atxn3 | -1.18916 | Down | 0.048174227 |
| Q8R0X7 | Sgpl1 | -1.16032333 | Down | 0.017751578 |
| Q99PG2 | Ogfr | -1.15791 | Down | 0.036999482 |
| P46662 | Nf2 | -1.15243333 | Down | 0.009802296 |
| Q99M08 |  | -1.11644333 | Down | 0.023800657 |
| Q3UN90 | Lyrm9 | -1.11275667 | Down | 0.001744003 |
| Q7M750 | Opalin | -1.09568333 | Down | 0.035898316 |
| P07759 | Serpina3k | -1.08241 | Down | 0.032288251 |
| Q8VHQ3 | Ppp1r16b | -1.0698 | Down | 0.003387775 |
| Q9QUI0 | Rhoa | -1.06848 | Down | 0.021667842 |
| O08677 | Kng1 | -1.06058 | Down | 0.049773791 |
| P01867 | Ighg2b | -1.05745667 | Down | 0.039192198 |
| O09114 | Ptgds | -1.05071333 | Down | 0.038558778 |
| P58774 | Tpm2 | -1.04110333 | Down | 0.043424186 |
| Q60766 | Irgm1 | -1.02503667 | Down | 0.014812339 |
| Q8VHI6 | Wasf3 | -1.01903667 | Down | 0.003261449 |
| Q9R1Z8 | Sorbs3 | -1.00605333 | Down | 0.004260405 |
| P03921 | Mtnd5 | 1.01941 | Up | 0.038339892 |
| P42227 | Stat3 | 1.084356667 | Up | 0.011301681 |
| P84104 | Srsf3 | 1.167773333 | Up | 0.040217404 |
| O35566 | Cd151 | 1.17917 | Up | 0.02026939 |
| Q61361 | Bcan | 1.223666667 | Up | 0.00760012 |
| Q8CEK3 | Spinkl | 1.279896667 | Up | 0.028642582 |
| Q00PI9 | Hnrnpul2 | 1.340803333 | Up | 0.005579346 |
| Q4LDD4 | Arap1 | 1.34096 | Up | 0.022483338 |
| Q8K3W0 | Babam2 | 1.380083333 | Up | 0.008523226 |
| Q61908 | Cmc4 | 1.410376667 | Up | 0.021424715 |
| P55066 | Ncan | 1.421196667 | Up | 0.038583812 |
| O35900 | Lsm2 | 1.55855 | Up | 0.043574109 |
| Q08943 | Ssrp1 | 2.157623333 | Up | 0.014319674 |
| P11103 | Parp1 | 2.196116667 | Up | 0.034117354 |
| Q3UPC7 |  | 2.341663333 | Up | 0.022116777 |
| P23475 | Xrcc6 | 2.395856667 | Up | 0.020727671 |
| Q64523 | H2ac20 | 2.461606667 | Up | 0.039536562 |
| P49183 | Dnase1 | 2.469183333 | Up | 0.020448887 |
| O55201 | Supt5h | 2.868963333 | Up | 0.0156334 |
| P24526 | Pmp2 | 3.583983333 | Up | 0.012976707 |
| O55103 | Prx | 3.724216667 | Up | 0.01707563 |
| O08716 | Fabp9 | 4.01288 | Up | 0.010396885 |
| P27573 | Mpz | 4.182516667 | Up | 0.00328843 |
| Q64237 | Dbh | 4.341586667 | Up | 0.00193421 |
| Q8R0F5 | Rbmx2 | 4.463856667 | Up | 0.010655781 |
| Q8BFQ4 | Wdr82 | 4.570493333 | Up | 0.000376823 |
| Q9DBE9 | Ftsj3 | 4.662496667 | Up | 0.048265886 |
| Q9JIK5 | Ddx21 | 4.80118 | Up | 0.01979739 |
| Q8C0I1 | Agps | 4.950106667 | Up | 0.000223303 |
| Q8R0A0 | Gtf2f2 | 4.965756667 | Up | 0.000167868 |
| Q9CQW9 | Ifitm3 | 5.236346667 | Up | 0.033596303 |
| Q9EST4 | Psmg2 | 5.420606667 | Up | 0.001295095 |
| P63280 | Ube2i | 5.67129 | Up | 0.000446132 |
| P49290 | Epx | 5.737076667 | Up | 0.03447078 |
| P50172 | Hsd11b1 | 5.867493333 | Up | 9.5666E-05 |
| Q61398 | Pcolce | 5.932923333 | Up | 0.035841329 |
| P49718 | Mcm5 | 6.02442 | Up | 0.000842059 |
| Q99L48 | Nmd3 | 6.501276667 | Up | 0.000110594 |
| Q9R098 | Hgfac | 6.54299 | Up | 0.003812404 |
| Q61129 | Cfi | 6.619883333 | Up | 1.45056E-05 |
| Q8BHE8 | Maip1 | 6.771303333 | Up | 0.000284294 |
| Q08288 | Lyar | 7.841083333 | Up | 0.000425724 |
